# Transcriptional rewiring of the GcrA/CcrM bacterial epigenetic regulatory system in closely related bacteria

**DOI:** 10.1371/journal.pgen.1009433

**Published:** 2021-03-11

**Authors:** Satish Adhikari, Ivan Erill, Patrick D. Curtis

**Affiliations:** 1 Department of Biology, University of Mississippi, University, Mississippi, United States of America; 2 Department of Biological Sciences, University of Maryland Baltimore County, Baltimore, Maryland, United States of America; Michigan State University, UNITED STATES

## Abstract

Transcriptional rewiring is the regulation of different target genes by orthologous regulators in different organisms. While this phenomenon has been observed, it has not been extensively studied, particularly in core regulatory systems. Several global cell cycle regulators are conserved in the Alphaproteobacteria, providing an excellent model to study this phenomenon. First characterized in *Caulobacter crescentus*, GcrA and CcrM compose a DNA methylation-based regulatory system that helps coordinate the complex life cycle of this organism. These regulators are well-conserved across Alphaproteobacteria, but the extent to which their regulatory targets are conserved is not known. In this study, the regulatory targets of GcrA and CcrM were analyzed by SMRT-seq, RNA-seq, and ChIP-seq technologies in the Alphaproteobacterium *Brevundimonas subvibrioides*, and then compared to those of its close relative *C*. *crescentus* that inhabits the same environment. Although the regulators themselves are highly conserved, the genes they regulate are vastly different. GcrA directly regulates 204 genes in *C*. *crescentus*, and though *B*. *subvibrioides* has orthologs to 147 of those genes, only 48 genes retained GcrA binding in their promoter regions. Additionally, only 12 of those 48 genes demonstrated significant transcriptional change in a *gcrA* mutant, suggesting extensive transcriptional rewiring between these organisms. Similarly, out of hundreds of genes CcrM regulates in each of these organisms, only 2 genes were found in common. When multiple Alphaproteobacterial genomes were analyzed bioinformatically for potential GcrA regulatory targets, the regulation of genes involved in DNA replication and cell division was well conserved across the Caulobacterales but not outside this order. This work suggests that significant transcriptional rewiring can occur in cell cycle regulatory systems even over short evolutionary distances.

## Author summary

The degree to which genetic or physiological systems evolve over evolutionary distance is often untested. One can assume that the same system in different organisms will change very little if 1) the evolutionary distance between the organisms is small, 2) the systems perform critical functions, and 3) the organisms have been under similar selective pressures (i.e. the organisms inhabited the same ecological niche). The Alphaproteobacteria offer an excellent opportunity to test this assertion as several critical global transcriptional regulators are conserved throughout this clade. In this study, the regulons of two such global regulators, GcrA and CcrM, in two closely related Alphaproteobacteria that inhabit the same ecological niche were compared and it was found that they regulate vastly different genes. In many cases, genes were present in both organisms, but targeted by a regulator in one organism and not in the other. These results suggest that significant transcriptional rewiring can occur even in a core regulatory system over small evolutionary distances and indicate that conservation of genes and genetic regulators may not be a complete indicator of their physiological function in an organism.

## Introduction

Bacterial global regulators can regulate the activity of dozens, if not hundreds, of genes. It is generally assumed that orthologous global regulators in closely related bacteria regulate similar sets of genes even when the organisms occupy different niches [1]. While this assumption is supported by a few cross organismal studies [2,3], regulon comparison has not been extensively performed. Regulon comparison is important because it can reveal how transcriptional regulatory circuits evolve over the time.

There are four different mechanisms by which regulatory circuits may evolve [1,4]. These include (1) embedding horizontally acquired genes under the regulation of transcription factor, (2) rearrangement of the orientation and/or position of the binding site with respect to transcriptional start site (promoter remodeling), and (3) changes in the transcription factor itself. The fourth (4) mechanism of regulatory circuit evolution is the gain or loss of transcription factor binding sites in the target promoters, such that the orthologous regulators have different regulatory targets in different organisms. This fourth mechanism is referred to as “transcriptional rewiring” [1]. Transcriptional rewiring has not been well-studied, and the majority of studies that have been performed have been in eukaryotic systems, particularly in yeast [5,6]. There have been only a handful of studies on transcriptional rewiring performed in prokaryotes [2,3].

Thus far in bacteria, transcriptional rewiring studies have focused primarily on metabolic regulatory systems, such as galactose metabolism [5], arabinose metabolism [2], or anaerobiosis [6]. In one study, minimal transcriptional rewiring was found when the AraC regulons were compared between *E*. *coli* and *Salmonella enterica* [2]. In another study, the FNR regulons were compared between the closely related Alphaproteobacteria *Rhodobacter capsulatus* and *Rhodobacter spaeroides*, as well as the distantly related *E*. *coli* [3]. As expected, FNR regulons were quite similar between the two *Rhodobacter* species with a small amount of transcriptional rewiring, but significantly different than that of *E*. *coli*, suggesting that transcriptional rewiring correlates with evolutionary distance.

The Alphaproteobacteria offer a perfect testbed to examine the evolution of cell cycle regulation. Several genes involved in *Caulobacter crescentus* developmental cell cycle regulation are well conserved across the entire Alphaproteobacteria clade [7]. These include *dnaA*, *gcrA*, *ccrM*, and *ctrA*. The only comparative studies performed in these systems examined CtrA [8–14]. In *C*. *crescentus*, CtrA is the master regulator of *C*. *crescentus* development and regulates cell division, chromosome replication, flagellum biosynthesis, chemotaxis, pilus production, and adhesion in that organism [15]. The CtrA regulon was identified in *Sinorhizobium meliloti* and included several of the same regulatory targets, such as motility, chemotaxis, and pili synthesis [8]. The CtrA regulon of a more distantly related Alphaproteobacterium, *Magnetospirillum magneticum* was identified and the only genes in common with the other identified CtrA regulons belonged to flagellum biosynthesis, suggesting that flagellum biosynthesis regulation was the ancestral role of CtrA, and other roles were acquired later in different Alphaproteobacteria [9].

One system that has not been examined across multiple organisms is the GcrA/CcrM system identified in *C*. *crescentus* [16,17]. The developmental cell cycle of *C*. *crescentus* is regulated by a cascade of global regulators that coordinate and control multiple cellular activities. GcrA and CcrM work together as a bacterial epigenetic system that participates in this global regulator cascade [18,19]. The methyltransferase CcrM is cell cycle regulated in *C*. *crescentus* and is expressed only in the late predivisional stage, after the bulk of chromosome synthesis has already occurred [20]. Because the *C*. *crescentus* genome is replicated only once per cell cycle, the chromosome remains hemimethylated for a significant amount of time before CcrM is expressed and fully methylates the chromosome [17,21]. In addition, since chromosome replication starts from the origin and moves towards terminus, promoters near the origin remain hemimethylated significantly longer compared to those that are close to the terminus [22]. *C*. *crescentus* uses this unusual pattern of DNA methylation to regulate gene expression during the cell cycle. In fact, microarray studies done by Gonzalez *et al*. (2014) showed that CcrM methylation impacts the expression of more than 10% of all *C*. *crescentus* genes [23]. GcrA is a transcriptional activator that binds to methylated GANTC sites and is hypothesized to activate genes containing a subset of such sites with the consensus sequence YGAKTCK within their promoter [18,19]. GcrA uses DNA methylation to control gene expression as a timing mechanism, coordinating gene expression with the progression of chromosome replication. More than 100 genes are misregulated in *gcrA* disruption strains [16,19] and, while there are diverse regulatory targets, a number of those genes are involved in chromosome replication and cell division.

In this study, the GcrA and CcrM regulons of *Brevundimonas subvibrioides* were identified and compared to *C*. *crescentus*. These bacteria live in the same freshwater environments, and in fact, both *C*. *crescentus* (CB15) and *B*. *subvibrioides* ATCC 15264 were isolated from the same pond, though in different years [24]. *B*. *subvibrioides* is a member of the *Caulobacteraceae* family, and thus even more closely related to *C*. *crescentus* than the *S*. *melitoti* strain used in the CtrA study, which is in a different order [25]. Both *Brevundimonas* and *Caulobacter* are very closely related genera within the *Caulobacteraceae* family with 16S rDNA similarities of 95–96% [26]. The *B*. *subvibrioides* and *C*. *crescentus* genomes share over 2000 orthologs and an average nucleotide identity of 74% [27]. *B*. *subvibrioides* has an asymmetrical cell cycle and produces two morphologically different daughter cells: a motile swarmer cell and a sessile cell similar to *C*. *crescentus*, suggesting cell cycle processes in both these bacteria are similar, including methylation state of the chromosome and cell cycle regulation of CcrM (although this has not been experimentally verified) [25]. In this study, global gene expression analysis was used to identify the GcrA and CcrM regulons in *B*. *subvibrioides* and to compare them with the *C*. *crescentus* regulons. According to previous regulon comparison studies, one would predict that the regulons would be very similar. However, the results presented here suggest significant divergence of these regulons driven by extensive transcriptional rewiring despite the small evolutionary distance between *B*. *subvibrioides* and *C*. *crescentus*. Our results hence demonstrate that regulatory systems, even ones critical to cell function, can diverge greatly through transcriptional rewiring.

## Results

### Identification of methylation motifs using SMRT sequencing in *B*. *subvibrioides*

To begin examining gene regulation by the GcrA/CcrM system in *B*. *subvibrioides*, DNA methylation was directly analyzed. While it has been previously shown that insertional disruption of the *B*. *subvibrioides ccrM* gene leads to phenotypic effects [25], expression of *ccrM* had not been analyzed and actual methylation of DNA by *B*. *subvibrioides* CcrM had not been directly verified. Additionally, *B*. *subvibrioides* has five other potential methyltransferases encoded in its genome [25]. To characterize the methylome of *B*. *subvibrioides*, Single Molecule Real Time (SMRT) sequencing was employed [28,29]. SMRT sequencing is a powerful technology that can directly detect N6-methyladenine as well as N4-methylcytosine in the DNA sequencing process; the sample DNA must undergo TET1 conversion to detect N5-methylcytosine, which was not performed in this study. Using SMRT sequencing to re-sequence the *B*. *subvibrioides* genome, N6-methyladenines were detected throughout the chromosome but no N4-methylcytosines were detected. Motif analysis was performed on sequences surrounding N6-methyladenines and a total of 7 motifs were detected (S1 Data). Out of those 7 motifs, one matched the CcrM motif G**A**NTC (methylated base in bold) which is also identified in *C*. *crescentus*. To verify that CcrM is responsible for the detected motif, SMRT sequencing was performed on the *ccrM*::pNPTS139 strain and the GANTC methylation motif was not detected, demonstrating that the *B*. *subvibrioides* CcrM ortholog is expressed and methylates this motif. Given the fact that the *C*. *crescentus* CcrM and *B*. *subvibrioides* CcrM are 74% identical at the amino acid level [25], this result is not surprising. Furthermore, motif analysis of the *ccrM* disruption strain showed only two predicted motifs that were also predicted in the WT (S1 Data). The absence of other motifs in *ccrM* strain suggests some of those motifs present in the WT might be due to spurious CcrM activity or that the absence of CcrM might lead to repression of other methyltransferases. Given that there are only 3 adenine methyltransferases aside from *ccrM* predicted in the *B*. *subvibrioides* genome, the former scenario appears more likely. Combining the data generated from SMRT sequencing of both the wild-type and *ccrM* strains, as well as predictions from the REBASE database [30], different motifs and potential methyltransferases responsible for their methylation are presented in Table 1. Bresu_2693 encodes CcrM, which is an adenine methyltransferase with a now confirmed G**A**NTC recognition motif. Bresu_3035 encodes a likely N6-adenine methyltransferase and REBASE predicts its motif to be A**A**TT; this motif was also detected in this study (in both WT and *ccrM* strains). The remaining adenine motif **A**GGCMGYA (detected in both WT and *ccrM* strain) could not be conclusively linked to a methyltransferase but is likely the motif of one of the two remaining predicted adenine methyltransferases (Bresu_1408 or Bresu_1999). While the technique used here was not capable of detecting N5-methylcytosine, Bresu_0174 is a predicted N5-cytosine methyltransferase orthologous to CCNA_03741 in *C*. *crescentus*, which has been shown to methylate cytosine in the GG**C**GCC motif [29]. REBASE database predicts Bresu_2033 to be a cytosine methyltransferase with the motif CCGCGG. Since no N4-methylcytosine was detected, this enzyme is either an N5-cytosine methyltransferase or it is not expressed. Given that a previous study showed that the gene encoding this enzyme is essential (and likely participates in a restriction-modification system) [25], Bresu_2033 likely codes for a N5-cytosine methyltransferase.

**Table 1:** Methylation motifs in *B*. *subvibrioides*.

Motifs	Modification type	Candidate methyltransferase gene	Partner restriction endonuclease	Motif predicted by REBASE database	Remarks	% of Motifs methylated	# of Motifs methylated	# of Motifs in Genome
GANTC	m6A	Bresu_2693^a^	Absent	Yes	Confirmed in this study	0.99	7765	7800
AATT	m6A	Bresu_3035 ^a^	Absent	Yes	Detected in this study	0.929	2204	2370
AGGCMGYA	m6A	Bresu_1999 or Bresu_1408	Absent for both genes	No	Detected in this study	0.329	150	455
GGCGCC	m5C	Bresu_0174 ^a^	Absent	Yes	Not detected in this study, predicted motif for *C*. *crescentus* ortholog [29]			
CCGCGG	m5C	Bresu_2033	Bresu_2032	Yes				

^a^: Homolog present in *C*. *crescentus*

While SMRT sequencing was used here to detect methylation sites, in doing so it also effectively re-sequenced the *B*. *subvibrioides* genome. This new genomic analysis predicted 3900 GANTC sites (7800 GANTC sites in total when both strands were considered since GANTC is palindromic) in the genome, compared to the 3899 GANTC sites predicted by the reference genome. The extra methylation site was found at genomic coordinates 2445157 to 2445161. Using the IPD ratio, CcrM recognition sites in *B*. *subvibrioides* were analyzed for their methylation status. Interpulse duration (IPD) ratio is a metric used in SMRT sequencing to identify methylated bases [28]. If the IPD ratio is greater than 1 for a particular base position, then it means that the polymerase slowed down at that particular position relative to the control, suggesting that some sort of modification is present on the template strand (methylation in this case). Out of 7800 GANTC sites (when both strands were considered), 7765 GANTC sites were found with adenine methylation and only 35 GANTC sites did not have methylation on their adenines. Upon closer inspection, 16 of these sites were found to be unmethylated on both strands (S1 Data) and 19 were found to be unmethylated only on one of the two strands (S1 Data). While SMRT sequencing was performed on DNA from unsynchronized *B*. *subvibrioides* cells, meaning chromosomes were likely under different stages of replication, the methylation status of individual sites is based upon the consensus methylation status of multiple reads over a given site in different DNA molecules. That is, those 19 GANTC sites with unmethylated adenines in only one of the strands should not be confused with hemimethylation that occurs during S-phase where the newly synthesized DNA is yet to be methylated by CcrM. Similar results have been found in *C*. *crescentus*, where 27 GANTC sites remained unmethylated throughout the cell cycle [29]. One potential explanation for this might be due to binding of another protein in the vicinity of the GANTC sites, thereby preventing access for CcrM. In *C*. *crescentus*, MucR1/2 proteins have been found to bind to at least some of these unmethylated sites and were also involved in the regulation of genes in the vicinity [31]. No consensus motif for MucR1/2 proteins has been identified in *C*. *crescentus*. MEME analysis was performed on the unmethylated sites (fifty bases upstream and downstream) to identify the potential DNA binding motifs which might prevent access but did not identify a consensus sequence. There is one ortholog of MucR1/2 in *B*. *subvibrioides* (Bresu_1201). However, when the genes in the vicinity of the unmethylated GANTC sites in *B*. *subvibrioides* were compared to the genes in the vicinity of unmethylated GANTC sites in *C*. *crescentus*, not a single gene was in common among them.

### Role of CcrM methylation in gene expression in *B*. *subvibrioides*

**Fig 1:**
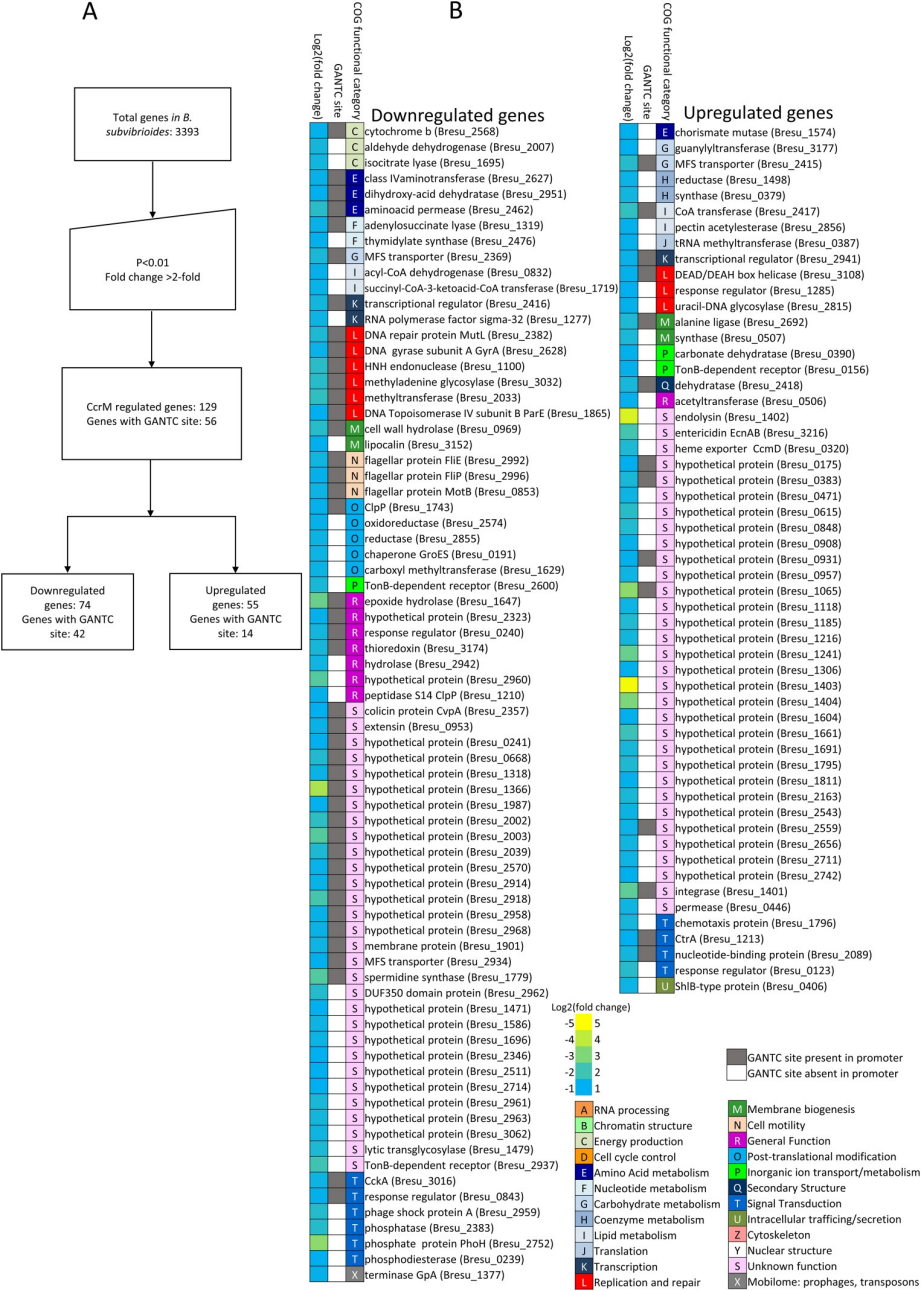
Genes misregulated in *ccrM* mutant compared to wild-type in *B*. *subvibrioides*. A) Workflow showing the cutoffs used for defining misregulated genes in *ccrM* mutant. Using >2-fold and P<0.01 as cut offs, 129 genes were found misregulated compared to WT out of 3393 total genes in *B*. *subvibrioides*. Out of 129 misregulated genes, 56 of them had at least one GANTC site in their promoter suggesting potential direct regulation by CcrM. B) List showing genes downregulated (left) and upregulated (right) in the *ccrM*::pNPTS139 strain along with COG functional category. For both left and right, Column 1 shows the heat map of the magnitude of fold change in log2 scale. Column 2 shows if those genes have GANTC site within their promoter (grey—GANTC site present, white—GANTC site absent). Genes were clustered by COG functional category (Column 3).

Previous research had shown that disruption of *ccrM* in *C*. *crescentus* was conditionally lethal (particularly when grown in PYE media [20]), while disruption of *ccrM* in *B*. *subvibrioides* resulted in no growth defect, suggesting a significant difference in the role of CcrM between these two organisms [25]. To begin studying the role of GANTC methylation in *B*. *subvibrioides* global gene expression, the expression profiles of wild-type and *ccrM* strains were compared using RNA-seq. Previous global gene expression studies of *ccrM* mutants *in C*. *crescentus* used a statistical cutoff of P<0.01 which resulted in 388 genes being characterized as misregulated [23]. When that same cutoff was applied to the *B*. *subvibrioides* RNA-seq data generated here, 1082 genes were characterized as misregulated, which is roughly a third of the genome. To make the dataset more specific, another cutoff of >2-fold change (compared to WT) in addition to P<0.01 was added. Based on these cutoffs, 129 *B*. *subvibrioides* genes were found to be misregulated in the *ccrM* mutant (Fig 1). To verify the RNA-seq data, the expression levels of 10 misregulated genes were analyzed by RT-qPCR, and all results matched the RNA-seq data except for *ctrA*, which showed a 2-fold increase in expression in the RNA-seq data but showed a decrease in expression by RT-qPCR (S1 Fig (bottom)). Out of the 129 misregulated genes, 74 were downregulated (Fig 1B (left) and S2 Data) in the *ccrM*::pNPTS139 strain and 55 were upregulated (Fig 1B (right) and S2 Data).

Given the nature of the RNA-seq technique as a global analysis method, and the fact that methylation plays a role in the global regulatory cascade, it is likely that a number of the misregulated genes are indirect regulatory targets of CcrM. To identify potential direct regulatory targets of CcrM, the promoter regions for each of the 129 genes were examined for methylation sites up to 200 bp upstream from the start codon. For genes that appeared to be in operons, the promoter region of the first gene of the operon was considered. This analysis revealed 51 promoter regions (covering 56 genes) that met the differential regulation cutoffs and had at least one GANTC motif in their promoter region. Out of these 56 genes, 42 genes were downregulated (Fig 1B and Table C in S1 Text) whereas 14 genes were upregulated (Fig 1B and Table D in S1 Text). Given the presence of 3900 methylation sites in the genome, one could expect that the presence of a methylation site in a promoter region would occur randomly with high probability, though a previous study by Gonzalez *et al* (2014) found that GANTC sites were overrepresented by at least 1.5-fold in intergenic regions across all Alphaproteobacteria except the *Rickettsiales* [23]. The presence of a methylation site combined with measurable changes in gene transcription lends higher confidence that these genes are direct regulatory targets of CcrM methylation. Genes were clustered by COG category to determine if specific functions were over-represented in the dataset (Fig 1B). Such categories include genes involved in DNA replication and repair, such as DNA gyrase subunit A (*gyrA*), DNA mismatch repair (*mutL*) and DNA topoisomerase IV subunit B (*parE*). Other categories include genes involved in cell motility, particularly in flagellar synthesis, such as *fliP*, *motB* and *fliE*. Important developmental signal transduction genes such as *cckA* and *ctrA* were also identified as potential direct CcrM targets.

### Regulatory targets of CcrM methylation in *B*. *subvibrioides* differ significantly from those in *C*. *crescentus*

**Fig 2:**
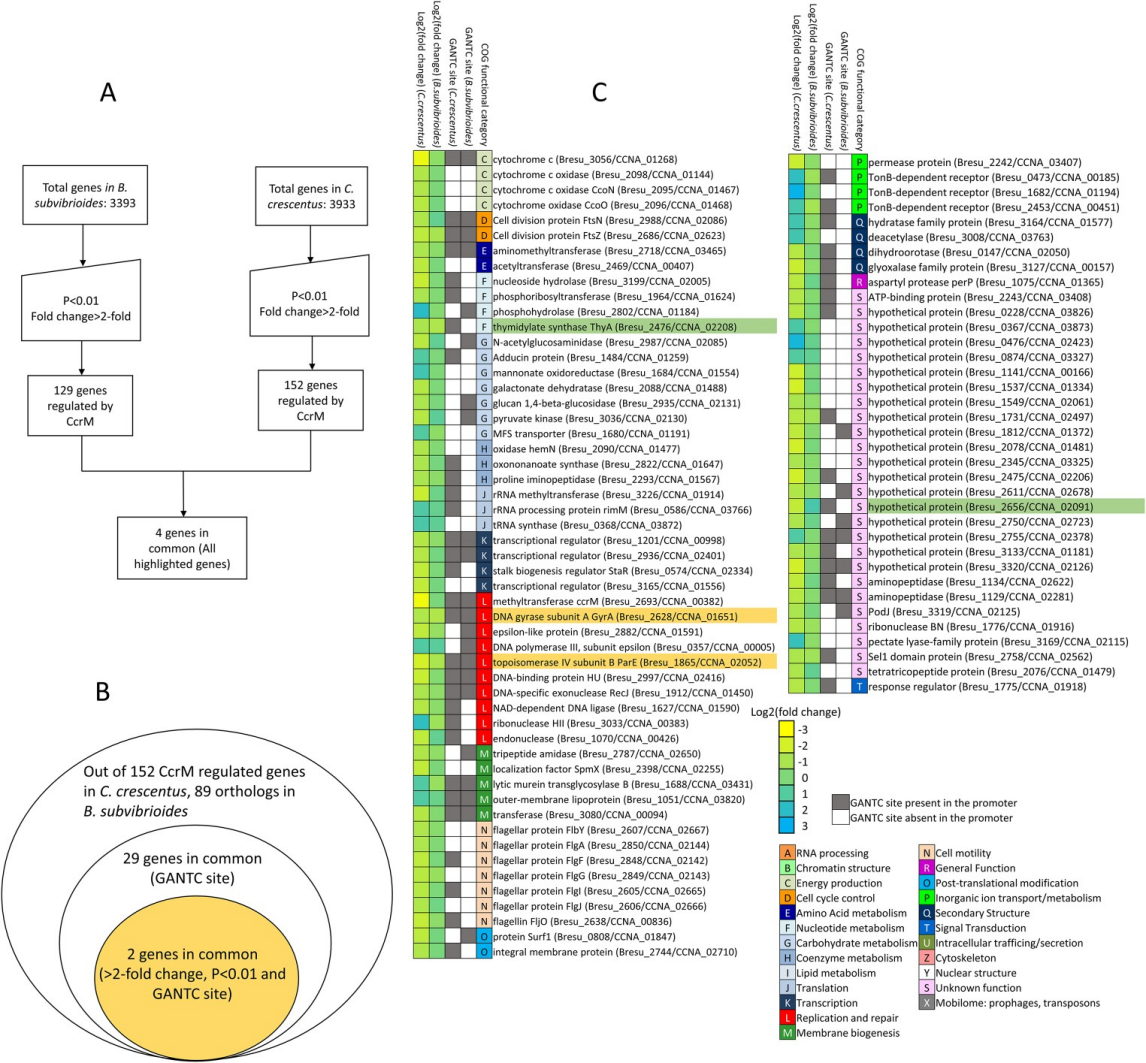
Common genes misregulated in *ccrM* mutant in *B*. *subvibrioides* and *C*. *crescentus*. A) Workflow showing the cutoffs used for defining misregulated genes in *ccrM* mutant in both organisms. Using >2-fold and P<0.01 as cut offs, 129 genes and 152 genes were found misregulated in *ccrM* in *B*. *subvibrioides* and *C*. *crescentus* respectively. Only 4 genes were found in common. B) Concentric circle diagram showing common genes using different parameters. *C*. *crescentus* CcrM regulates 152 genes and *B*. *subvibrioides* has orthologs to 89 of those genes. Only 29 of the *B*. *subvibrioides* orthologs have GANTC sites in their promoter regions. Of those 29, only 2 genes showed significant transcriptional changes in a *ccrM* mutant strain (highlighted in orange). C) List of 89 *B*. *subvibrioides* orthologs to *C*. *crescentus ccrM* regulated genes sorted by COG functional category. Column 1 and 2 shows the heat map of the magnitude of fold change in log2 scale in the *C*. *crescentus ccrM* strain (data obtained from [23]) and *B*. *subvibrioides ccrM*::pNPTS139 strain respectively. Columns 3 and 4 show if those genes have GANTC site within their promoter in *C*. *crescentus* and *B*. *subvibrioides* respectively (grey—GANTC site present, white—GANTC site absent). Genes were clustered by COG functional category (Column 5). Orthologs that met the transcriptional change cutoffs are highlighted in green; orthologs that met the transcriptional change cutoffs and have a GANTC site are highlighted in orange.

Previous global gene expression analysis in *C*. *crescentus* using microarrays found that 388 genes were misregulated in *ccrM* mutant compared to WT using P<0.01 as a cut off [23]. As described above, this cutoff is too permissive to realistically analyze the *B*. *subvibrioides* data. In order to make the *C*. *crescentus* dataset comparable to the *B*. *subvibrioides* dataset, the same two-parameter cutoff (P<0.01 and >2-fold change) was applied to the previously published *C*. *crescentus* dataset, resulting in 152 genes characterized as misregulated in the *C*. *crescentus ccrM* mutant [23]. When the 152 genes misregulated in *C*. *crescentus ccrM* (P<0.01 and >2-fold change) were compared to the 129 genes misregulated in *B*. *subvibrioides ccrM* (P<0.01 and >2-fold change) only 4 genes were in common (Fig 2A and 2C, and Table E in S1 Text). This comparison included both direct and indirect regulatory targets. When the presence of a methylation site in the promoter region (+200 bp from start codon) was added as a criterion to compare direct regulatory targets, only 2 genes were in common (Fig 2B and genes highlighted in orange Fig 2C). The almost complete lack of regulon conservation is surprising given how closely related the two organisms are. Two factors appear to contribute to the lack of conservation. First is the loss of gene content. Of the 152 genes misregulated in *C*. *crescentus ccrM* (P<0.01 and >2-fold change), *B*. *subvibrioides* has orthologs for 89 of them (Fig 2B and all genes listed in Fig 2C). Out of 129 misregulated genes in *B*. *subvibrioides ccrM* (P<0.01 and >2-fold change), *C*. *crescentus* has orthologs for 80 of them. Second is transcriptional rewiring, as indicated here by the loss of methylation sites. Of the 89 *B*. *subvibrioides* orthologs, only 29 of them have methylation sites in the promoter regions (Fig 2C, GANTC column). Finally, only 2 of those 29 genes show significant transcriptional changes in a *ccrM* mutant (genes highlighted in orange in Fig 2C).

It is possible that the cutoffs used for comparison were too stringent. However, taking the 388 genes misregulated in the *C*. *crescentus ccrM* mutant (P<0.01) and comparing them to 129 genes from *B*. *subvibrioides* (P<0.01 and >2-fold) only resulted in 12 common genes. Relaxing the *B*. *subvibrioides* cut off to P<0.01 and >1.8-fold only gave 17 genes in common. These results suggest that the lack of regulon conservation is not an artefact of cutoff choice and instead reflects real divergence between these regulons. It should be noted that the *C*. *crescentus* study was performed using M2G minimal medium to bypass the lethality of *ccrM* disruption, but PYE medium was used in this study because *B*. *subvibrioides* does not grow in M2G medium. It is unclear what effect growth media has on the results, but other regulon comparison studies using different media [3,8] have only identified limited transcriptional rewiring.

### Identification of regulatory targets of GcrA in *B*. *subvibrioides*

**Fig 3:**
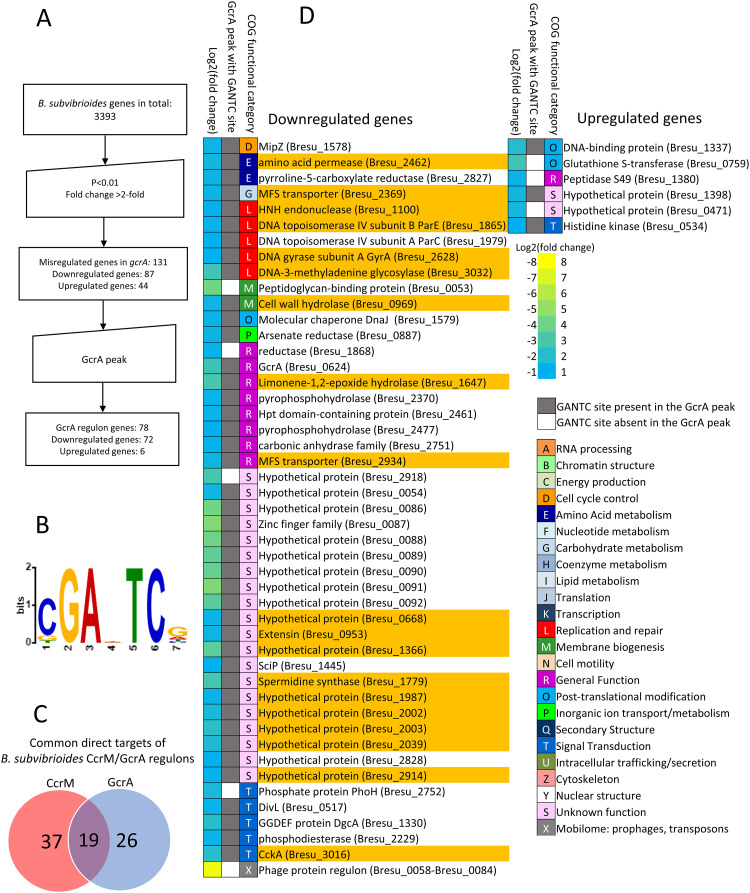
Genes directly regulated by GcrA in *B*. *subvibrioides*. A) Workflow showing the cutoffs used for defining the GcrA regulon. Using transcriptional change cutoffs of >2-fold and P<0.01, 131 genes were characterized as misregulated in the *gcrA* mutant. Of those 131 genes, GcrA peaks (obtained from ChIP-seq) were detected in the promoter regions of 78 genes. B) MEME analysis of promoters activated by GcrA that had only one GANTC site within the promoter region in *B*. *subvibrioides*. In total, 18 genes were activated by GcrA with only one GANTC site within the promoter region in *B*. *subvibrioides*. MEME analysis showed no preference for any bases in an extended GANTC motif beyond a slight preference for C before GANTC site. C) Venn diagram showing common genes of CcrM/GcrA regulons in *B*. *subvibrioides*. There were 56 genes directly regulated by CcrM (>2-fold, P<0.01 and GANTC site in promoter), and 45 genes directly regulated by GcrA with methylation sites (>2-fold, P<0.01 and GcrA peak with GANTC site in promoter). Only 19 genes were found in common between them (highlighted in orange in Fig 3D). D) List showing all misregulated genes with GcrA peaks (with or without GANTC site) that were downregulated (left) and upregulated (right) in the Δ*gcrA* strain sorted by COG functional category. For both left and right, Column 1 shows the heat map of the magnitude of fold change in log2 scale. Column 2 shows if GANTC site is also present in the GcrA peak (grey—GANTC site present, white—GANTC site absent. Genes were clustered by COG functional category (Column 3).

While CcrM-dependent methylation clearly affects gene transcription, it is not believed that methylation directly alters transcription. Rather, methylation has been postulated to alter the binding and/or activity of the regulatory protein GcrA [32]. To begin characterizing the GcrA regulon in *B*. *subvibrioides*, RNA-seq was performed comparing gene expression between a *B*. *subvibrioides gcrA* mutant and the wild type. Once again, using the statistical P<0.01 and >2-fold change in expression cutoffs, 131 genes were characterized as misregulated in the *gcrA* mutant. To verify the RNA-seq data, the expression levels of 10 misregulated genes were analyzed by RT-qPCR, and all the results matched the RNA-seq data (S1 Fig (Top)). Out of the 131 misregulated genes, 87 genes were downregulated while 44 genes were upregulated (Fig 3A and S3 Data) in the *gcrA* mutant compared to WT.

In *C*. *crescentus*, GcrA affects the production of the next global regulator in the developmental cascade, CtrA. Therefore, it is likely that a number of genes with altered transcription are indirect targets of GcrA. To better assess GcrA’s direct regulatory targets in *B*. *subvibrioides*, ChIP-seq was performed to identify genomic areas directly bound by GcrA and combined with RNA-seq to identify genes under direct regulation of GcrA. Using ChIP-seq, 879 GcrA binding peaks (S4 Data) were identified that were significantly enriched compared to the input DNA (DNA from same samples before IP) with very high confidence (3 replicates with correlation of 0.95). About half of these peaks were found in intergenic regions with roughly equivalent numbers found in intragenic regions. These results are similar to what has been found in *C*. *crescentus* [19].

It is thought that instead of GcrA binding to a target DNA sequence and then recruiting σ^70^, GcrA binds to σ^70^ and stimulates transcriptional activity when σ^70^ binds to a promoter with an adjacent methylation site. In such a scenario, it is possible that many promoters that bind to σ^70^ might also be pulled down along with GcrA, even though they do not have a GANTC site in their promoter and thus are not transcriptionally impacted by GcrA. However, multiple attempts were made to perform ChIP-seq in a *ccrM* mutant background and none yielded enough DNA to perform sequencing, suggesting promiscuous pulldown of DNA through a GcrA/σ^70^ interaction is not a significant contributor to the GcrA ChIP-seq dataset. Additionally, it is not clear why there were so many GcrA peaks (>400) found in the coding region of genes. One possibility is that free GcrA not interacting with σ^70^ may still bind to GANTC sites including those in coding regions. However, there are thousands of methylation sites that were not bound by GcrA. What exactly governs GcrA binding and/or transcription regulation is still not clear.

In *C*. *crescentus*, the GcrA regulon was defined as genes with >1.75-fold lower expression compared to WT and a detectable peak -40 bp to +40 bp from the transcriptional start site because those criteria explained most of the data [19]. However, global transcriptional start site data is unavailable for *B*. *subvibrioides* so here the scope of promoter region was broadened. Genes under the direct regulation of GcrA in *B*. *subvibrioides* were categorized as having >2-fold change in expression in the *gcrA* mutant compared to wild-type with P<0.01 and the presence of a GcrA peak in the promoter region, here defined as -100 bp to +100 bp of the translational start site (Fig 3A). The presence of a GANTC site as an additional criterion to define the GcrA regulon in *B*. *subvibrioides* was not included because not all GcrA peaks included a methylation site (Fig 3D), unlike the previous *C*. *crescentus* study where this criterion was included. For those genes that belonged to an operon, the promoter region of the first gene was considered. Using these criteria, 78 genes were characterized as being under direct regulation of GcrA (Fig 3). Out of these, 72 genes were found to be downregulated (Fig 3D and Table F in S1 Text) in the *gcrA* mutant whereas only 6 genes were found to be upregulated (Fig 3D and Table G in S1 Text). These data are consistent with findings in *C*. *crescentus* that suggest GcrA acts principally (or even solely) as an activator [19].

As before, genes were clustered by COG category to determine if certain functions were over-represented in the dataset (Fig 3D). Similar to the CcrM regulon, genes involved in DNA replication and repair were found in greater numbers, including the same *gyrA* and *parE* genes as seen in the CcrM regulon, as well as *mipZ* (cell cycle control and cell division). Signal transduction genes also had increased representation, such as *cckA* (CtrA activation). Included in the dataset was one large 26 gene operon (Bresu_0058-Bresu_0084). Protein BLAST of this region showed several hypothetical proteins along with a prophage tail length protein, peptidase U35 phage prohead protein, phage portal protein, and terminase GpA, strongly suggesting this region belongs to a prophage. Interestingly, this entire operon is expressed in the wild-type cells in a GcrA-dependent manner. No phage particles have been observed in wild-type cultures imaged by TEM [33], suggesting that even if this operon constitutes a prophage, it is non-functional. One explanation for the results is that the phage genome excised in the *gcrA* disruption strain, but when this strain was analyzed by PCR, the phage genes were still present. It is not clear why this operon would be regulated by GcrA, but it is notable that the *C*. *crescentus* phage Phi-CbK does contain a GcrA homolog in its genome [34]. It has been speculated that Phi-CbK may express its own GcrA as a mechanism of stalling the *C*. *crescentus* cell cycle to better redirect resources towards phage production.

Surprisingly, one of the genes characterized as being under direct regulation of GcrA in *B*. *subvibrioides* was *sciP*, an important regulator of CtrA activity. In *C*. *crescentus*, *sciP* expression is activated by CtrA not GcrA [35]. GcrA regulation of *sciP* would constitute a significant change to the cell cycle. While the promoter region of *sciP* in *B*. *subvibrioides* contains a GcrA binding site, it also has a CtrA binding site and therefore it is possible that the decreased expression of *sciP* in the *gcrA* strain was due to an indirect regulatory effect of decreased *ctrA* expression (decreased *ctrA* expression in the *gcrA* strain was seen in the RNA-seq data). Mutation of the CtrA binding site abolished *sciP* transcription in both the WT and *gcrA* strains, indicating that GcrA is not capable of driving expression on its own, and that the reduction in *sciP* expression in the *gcrA* mutant is an indirect effect of decreased *ctrA* expression (S2 Fig).

Previous research in *C*. *crescentus* has led to the hypothesis that gene activation by GcrA occurs only for methylated GANTC sites that have the extended methylation motif TGATTCG or more broadly, YGAKTCK [19,32]. To examine if GcrA favored binding to an extended motif in *B*. *subvibrioides*, genes activated by GcrA that had only one GANTC site (18 genes in total in *B*. *subvibrioides*) were analyzed by MEME (Fig 3B). No preference for any bases in an extended GANTC motif beyond a slight preference for C before the GANTC site was found.

The GrcA and CcrM regulons in *B*. *subvibrioides* were compared to identify the core regulatory targets of the GcrA/CcrM system in this organism. There are 56 genes in the CcrM regulon that show >2-fold change, P<0.01 and have a methylation site in the promoter region (Fig 1). There are 45 genes in the GcrA regulon that show >2-fold change, P<0.01, have a GcrA binding peak and a methylation site within that peak (Fig 3D). When those datasets were compared, 19 genes were in common (Fig 3C and 3D highlighted and Table H in S1 Text). Given the relationship between GcrA and methylation, this is perhaps less overlap in regulons than expected. For example, in *C*. *crescentus* the 204 genes regulated by GcrA were compared with 78 genes belonging to CcrM regulon (>2-fold change, P<0.01 and presence of GANTC site in promoter) and 33 genes in common (Table I in S1 Text). In *B*. *subvibrioides*, of the 56 presumed direct regulatory targets for CcrM, 37 of them appear to be regulated in a non-GcrA-dependent fashion. A potential explanation is the presence of other methylation dependent regulators in *B*. *subvibrioides*. Of note, 14 direct regulatory targets are upregulated in a *ccrM* mutant and none of them were found in the GcrA regulon, suggesting the presence of a methylation-dependent repressor. There were 26 genes misregulated in the *gcrA* mutant, with GcrA binding peaks and methylation sites in the promoter region, that were not part of the CcrM regulon (Fig 3D). It is possible that GcrA is still able to regulate these genes to a certain extent even if methylation is absent as it is, after all, only a small structural change to the binding site. Of the genes common to both regulons, genes involved in replication and repair were enriched compared to most other functional categories (Fig 3D). Conversely, while GcrA regulated a number of signal transduction genes, almost none of them (except for *cckA*) were found in the CcrM regulon.

### The GcrA regulon in *B*. *subvibrioides* differs from that of the *C*. *crescentus* GcrA regulon

In order to understand how the GcrA regulon has evolved in these bacteria, the GcrA regulon of *B*. *subvibrioides* was compared to that of *C*. *crescentus*. As mentioned in the introduction, there are four different mechanisms by which regulatory circuits may evolve. These mechanisms include changes in the transcription factor itself, promoter remodeling, embedding horizontally acquired genes and transcriptional rewiring. Given that GcrA in *C*. *crescentus* and *B*. *subvibrioides* are 68% identical at the amino acid level and MEME analysis showed that the binding motif of GcrA in *B*. *subvibrioides* is similar to that of *C*. *crescentus* (Fig 3C), it is likely that *B*. *subvibrioides* and *C*. *crescentus* GcrA operate in a similar manner. Additionally, 72% of the *C*. *crescentus* GcrA regulon genes have orthologs in the *B*. *subvibrioides* genome (see below), suggesting that horizontal gene transfer has not had a significant impact on differences between the regulons. The nature of the GcrA activation mechanism also indicates promoter remodeling is not a major factor. The GcrA binding site is essentially palindromic, so reorientation of the site will have little effect on transcriptional activation, and binding sites both upstream and downstream of promoters in *C*. *crescentus* have been found to activate transcription [19], so moderate repositioning of the binding site should not affect transcription substantially. Therefore, if changes are observed between the regulons of the two organisms, this is most likely due to transcriptional rewiring.

Haakonsen *et al*. (2015) used microarrays and ChIP-seq to identify the direct regulatory targets of GcrA in *C*. *crescentus* [19]. In that study, the chromosomal *gcrA* was deleted and a copy of the gene was expressed from an inducible vanillate promoter on the chromosome. The synchronizable NA1000 strain of *C*. *crescentus* was used and *gcrA* was pre-depleted for 30 mins before synchrony. A direct regulatory target of GcrA was defined as a gene with at least 1.75-fold lower expression compared to WT, with a GcrA peak, and at least one GANTC site in its promoter region (-40 bp to 40 bp from the transcriptional start site). Using these criteria, 204 genes were identified as being under the direct regulation of GcrA.

In order to compare the GcrA regulon between *C*. *crescentus* and *B*. *subvibrioides*, criteria similar to those of the Haakonsen *et al*. (2014) *C*. *crescentus* study were used. Because transcriptional start site profiling has not been performed in *B*. *subvibrioides*, the promoter region was defined as -100 to +100 bp from translational start sites. Though the presence of GANTC sites in the promoter region was not used as a criterion in the previous section (see above), it was included as a criterion here to better match the Haakonsen study. Also, to be consistent with the Haakonsen study, a cutoff of at least 1.75-fold lower expression compared to WT was used, and genes with higher expression in the *gcrA* mutant were also omitted.

**Fig 4:**
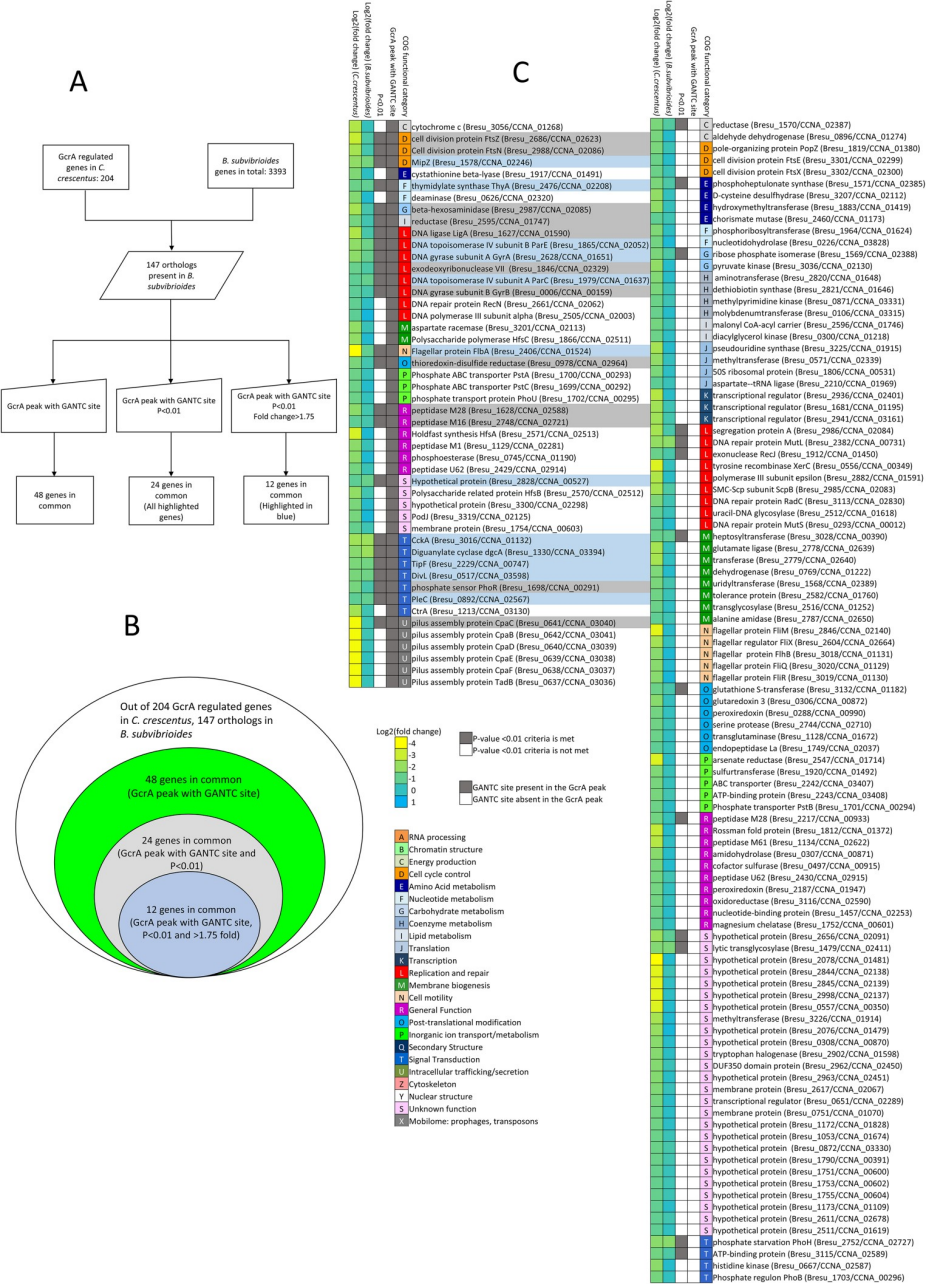
Common genes regulated by GcrA in *B*. *subvibrioides* and *C*. *crescentus*. A) Workflow showing the cutoffs used for defining common genes belonging to GcrA regulon in *B*. *subvibrioides* and *C*. *crescentus*. Out of 204 GcrA regulated genes in *C*. *crescentus*, 147 orthologs found in *B*. *subvibrioides*. Only few genes were in common despite using different cutoffs to define GcrA regulon in *B*. *subvibrioides*. B) Concentric circle diagram showing common genes using different cutoffs. *B*. *subvibrioides* has 147 orthologs to the 204 GcrA targets in *C*. *crescentus*. Of those 147 genes, only 48 had detectable GcrA peaks (obtained from ChIP-seq data) with GANTC sites. Only 24 of those 48 genes had transcriptional changes meeting a P<0.01 cutoff in the *gcrA* mutant (all highlighted genes in Fig 4C), and only 12 of those met the >1.75-fold change transcriptional cutoff (highlighted blue in Fig 4C). C) List showing all 147 *B*. *subvibrioides* genes orthologous to the 204 members of the published *C*. *crescentus* GcrA regulon, sorted by COG functional category. For both left and right, Column 1 and 2 is the heat map showing the magnitude of fold change in log2 scale in *C*. *crescentus gcrA* strain (data obtained from [19]) and *B*. *subvibrioides gcrA* strain respectively. Column 3 shows genes that met P<0.01 criteria or not in *B*. *subvibrioides* (grey—P<0.01 is met, white—P<0.01 is not met). Column 4 shows if those genes have GcrA peaks with GANTC sites within their promoter in *B*. *subvibrioides* (grey—GcrA peak with GANTC site present, white—GcrA peak with GANTC site absent). Genes were clustered by COG functional category (Column 5). Orthologs with GcrA peaks containing a GANTC site are shown in the left and orthologs without GcrA peaks containing a GANTC site are shown in the right. Orthologs with GcrA peak and P<0.01 are highlighted (blue and gray). Orthologs with GcrA peak, P<0.01 and >1.75-fold change are shown in highlighted blue.

Out of the 204 genes regulated by GcrA in *C*. *crescentus*, *B*. *subvibrioides* had orthologs for 147 (Fig 4). However, of those 147 genes, only 48 had GcrA peaks with a GANTC site in their promoter region (Fig 4A and 4C (left)). Therefore, without using transcriptional data, nearly two-thirds of the published *C*. *crescentus* GcrA regulon (99 genes) are clearly not part of the *B*. *subvibrioides* GcrA regulon despite being conserved in the genome, suggesting significant transcriptional rewiring has taken place. Applying a transcriptional change cutoff of P<0.01 reduces the number of common genes to 24 (All genes highlighted in Fig 4 and Table J in S1 Text). Adding a transcriptional change of >1.75 lower expression than WT reduces the number of common genes to 12 (Genes highlighted in blue in Fig 4). Given the important cell-cycle role of GcrA, this is a surprising lack of conservation. While loss of gene content is a contributor to regulon divergence, it appears that the major driving force behind the divergence is transcriptional rewiring, with nearly 100 orthologous genes in *B*. *subvibrioides* lacking GcrA binding sites. Applying the Haakonsen study cut-offs to the *B*. *subvibrioides* data results in 51 genes characterized as regulatory targets of GcrA in that organism. Of those 51 genes, *C*. *crescentus* has orthologs for 32 of them but 20 of them are not GcrA targets in *C*. *crescentus*, suggesting these genes have been transcriptionally rewired at some point. When common genes were analyzed by functional category, they were enriched in genes involved in replication and repair as well as signal transduction (Fig 4C), suggesting these could be core functional targets of GcrA.

### Bioinformatics analysis suggests genes involved in DNA replication, cell division and *ctrA* regulation are activated by GcrA within the order Caulobacterales

The experimental data presented above suggests that GcrA regulons have vastly different regulatory targets in *C*. *crescentus* and *B*. *subvibrioides*. In order to identify core (common) and auxiliary (species-specific) genes regulated by GcrA, a comparative genomics approach was implemented to analyze and compare putative GcrA regulons across different phylogenetic levels of the Alphaproteobacteria. First, potential GcrA regulatory targets were identified and compared in the closely related *Caulobacteraceae* and *Hyphomonadaceae* families that belong to the order Caulobacterales [36]. The analysis included the 23 available complete genomes in these families harboring a GcrA homolog. The presence of GcrA homologs in target genomes was determined via a BLASTP search restricted to the Caulobacterales, using the *B*. *subvibrioides* GcrA protein as query and with limiting e-value of 10^−20^ and query coverage of 75% (S5 Data). For each genome, protein coding genes were analyzed for the presence of at least one instance of the extended GANTC motif (YGAKTCK) within their promoter regions using a PSSM model of the extended GANTC motif. Promoter regions were defined as spanning from -200 bp to +100 bp of the start codon, irrespective of any other annotated features upstream of the start codon. The results of this search for extended GANTC sites across multiple genomes were aggregated for ortholog groups, as determined via reciprocal BLAST searches. As expected, due to their short length, extended GANTC motifs were identified upstream of many genes across all genomes. Hence, the mere presence of an extended GANTC motif instance is not an effective proxy of GcrA regulation. The conservation of these motif instances in the promoter regions of genes belonging to the same ortholog group among different organisms, however, could potentially be indicative of GcrA regulation, since the regulatory effect may be selected for and thus preserved across species. To test this hypothesis, 1,355 ortholog groups with orthologs in at least 20 of the 23 *Caulobacteraceae* and *Hyphomonadaceae* species and presenting one extended GANTC motif (YGAKTCK) instance in at least one of the target genomes were analyzed. Different metrics based on the score, conservation and number of identified extended GANTC sites upstream of genes in putatively regulated ortholog groups were evaluated by assessing their rank correlation with GcrA ChIP-seq enrichment scores for *C*. *crescentus* [19] and *B*. *subvibrioides*. The best correlation (*C*. *crescentus* ρ = 0.29, P<0.001; *B*. *subvibrioides* ρ = 0.25, P<0.001) was obtained for the inter-species average of best extended GANTC instance scores in their promoter region, weighted by the conservation and average number of sites per promoter: $\langle W_{S_{max}}\rangle =\langle s_{max}\rangle \frac{{Sp}_{site}}{{Sp}_{orth}}\langle \left|sites\right|\rangle$. This metric takes into account, for each ortholog group, the average maximum score of extended GANTC sites across species <*s*_*max*_*>*, the pervasiveness of extended GANTC sites across orthologs *Sp*_*site*_/*Sp*_*orth*_, computed as the ratio of the number of species presenting at least one extended GANTC site instances versus the number species encoding the ortholog, and the average number of extended GANTC sites across species *<*|*sites*|*>*. The $\langle W_{S_{max}}\rangle$ score therefore is high for genes presenting residual evidence of GcrA regulation across multiple species, a large average number of sites and a high average site score. High $\langle W_{S_{max}}\rangle$ scores may therefore be achieved by ortholog groups showing homogeneous, moderately high site number and score values, or by the consistent presence of large number of sites or very high scores in a few species that drive up the average values. For each ortholog group, we also assessed the posterior probability of regulation in each species, which combines the PSSM scores of all extended GANTC sites within a promoter region into a formal probability of regulation [37]. The posterior probability of regulation assumes that all sites contribute independently to the regulatory effect of GcrA and, therefore, is a function of the number and quality of the identified sites that does not consider specific location of sites in the promoter architecture.

**Fig 5:**
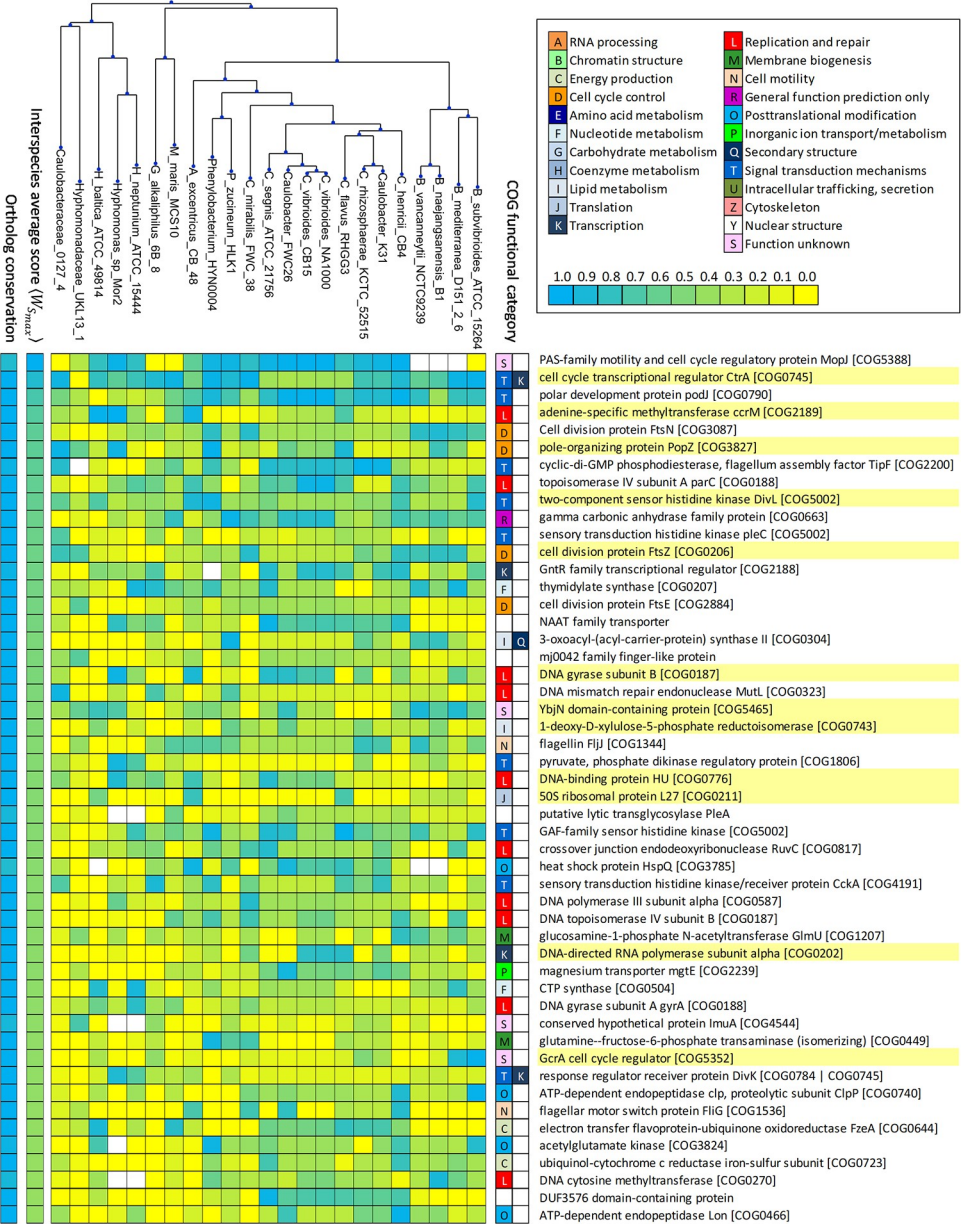
Heatmap showing top 50 ranked highly conserved ortholog groups for *Caulobacteraceae* and *Hyphomonadaceae* families. Each column designates a species, following the order dictated by a phylogenetic tree inferred from a multiple sequence alignment of GcrA homologs. Rows correspond to identified orthologous groups. For each cell, the cyan-yellow scale coloring indicates the posterior probability of regulation of the ortholog in that species (cyan blue—1, yellow—0). White cells indicate absence of the ortholog in that particular species. The left ancillary columns indicate, using the same color scale, the number of orthologs in each ortholog group (lowest value 20 out of 23) and the inter-species average of best extended GANTC instance score $\langle W_{S_{max}}\rangle$, which has been used to rank the ortholog groups. Both values are shown normalized to the (0,1) range. The right ancillary columns indicate the two primary functional categories for the COGs assigned to each ortholog group, the description and identifier of which is shown adjacent. Highlighted descriptions denote ortholog groups also present in Fig 6.

Ranking ortholog groups using the inter-species average of best extended GANTC instance scores $\langle W_{S_{max}}\rangle$, the top 50 highest scoring genes included 10 of the *B*. *subvibrioides* GcrA regulon members described here, such as *gyrA*, *parC*, *divL*, *cckA* (Fig 5). In addition, *B*. *subvibrioides* orthologs for 30 genes in this set met the criteria defined for either RNA-seq or ChIP assays and include several genes involved in cell-division such as *ftsN* or *ftsZ*. Among the genes not matching RNA-seq criteria are several SOS regulon members (e.g. *ruvC*, *imuA*) and other transcriptional regulators. The Alphaproteobacteria SOS repressor, LexA, targets a degenerate GTTC-N7-GTTC motif [38], which can easily overlap GANTC sites, explaining both their conservation and the lack of apparent regulatory effect due to the quasi-permanent occupancy of these regions by LexA.

It was remarkable that the 8 *Caulobacter* species showed similar posterior probabilities of regulation for many of the top 50 highest scoring genes, suggesting that the amount of transcriptional rewiring within the *Caulobacter* genus is rather limited. The most phylogenetically distant *Caulobacter* species, *C*. *mirabilis*, despite having a GcrA ortholog, appears to lack substantial evidence of regulation for many of the top 50 highest scoring genes (Fig 5). Among the top 50 highest scoring genes, several genes involved in DNA replication and repair, such as *parC* and *gyrB* were also found to be conserved across all 23 species, with relatively high posterior probability of being regulated by GcrA in most of the species (Fig 5). All these genes are part of GcrA regulon in *C*. *crescentus* and *B*. *subvibrioides*. This suggests that GcrA has a conserved role in regulating DNA replication and repair genes in the Caulobacterales. Several genes involved in cell division, such *as ftsN*, *ftsZ* and *ftsE* have really interesting patterns possibly attributable to transcriptional rewiring; it seems that *ftsN* and *ftsZ* are most likely to be regulated by GcrA specifically in *Brevundimonas* species (>0.7) and less so in closely related *Caulobacter* species (~0.3) and other Caulobacterales members (Fig 5). For *ftsN*, given the posterior probabilities for all the members of Caulobacterales, this data set seems to suggest that regulation of *ftsN* by GrcA could be newly acquired in the *Brevundimonas* species (Fig 5). This is in contrast to the pattern seen for *ftsE*, which exhibits moderate posterior probabilities (~0.5) for *Caulobacter* species and lower posterior probabilities in almost all other genera (Fig 5). The cell cycle regulator CtrA was identified as conserved in all the species and assigned a high posterior probability of regulation by GcrA in almost all of them (Fig 5). Another gene involved in CtrA regulation, *divL* was found in all the 23 species and likely to be regulated by GcrA in most of them (Fig 5). This gene belongs to GcrA regulon in *C*. *crescentus* and *B*. *subvibrioides* as well. In addition, the *podJ* gene, which is involved in cell differentiation and localizes to the swarmer pole in the predivisional cell, was also found likely to be under GcrA regulation in most of the species analyzed (Fig 5). These findings are consistent with the experimental data for *B*. *subvibrioides* and *C*. *crescentus* and point towards a GcrA regulon in the Caulobacterales comprising DNA replication and repair, cell division as well as signal transduction (particularly CtrA regulation).

**Fig 6:**
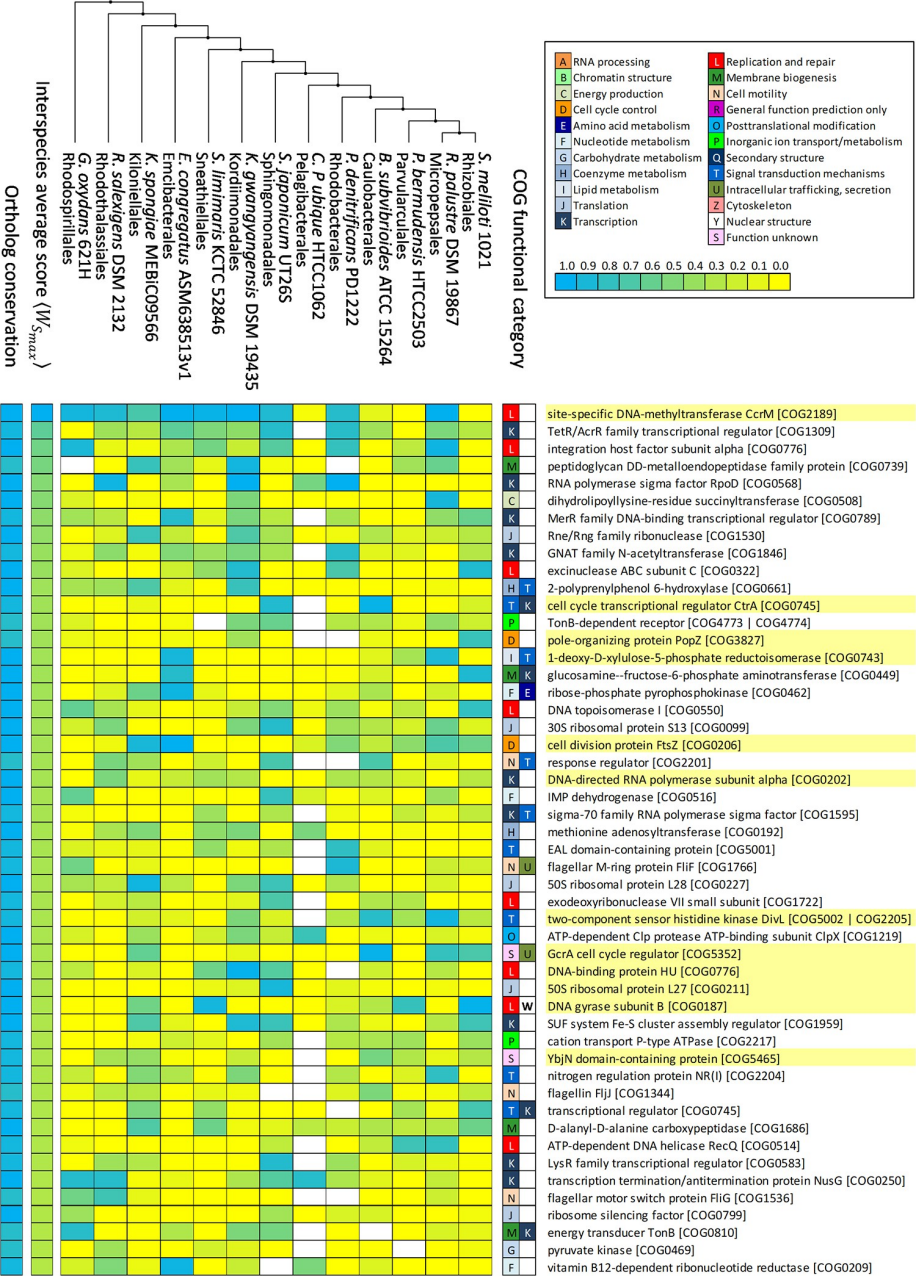
Heatmap showing top 50 ranked highly-conserved ortholog groups for representatives of each Alphaproteobacteria order. Each column designates a species, following the order dictated by a reference cladogram adapted from [44], placing *Emcibacter congregatus* and *Rhizomicrobium palustre* following their reported phylogeny in [45] and [46] respectively. Rows correspond to identified orthologous groups. For each cell, the cyan-yellow scale coloring indicates the posterior probability of regulation of the ortholog in that species (cyan blue—1, yellow—0). White cells indicate absence of the ortholog in that particular species. The left ancillary columns indicate, using the same color scale, the number of orthologs in each ortholog group (lowest value 11 out of 13) and the inter-species average of best extended GANTC instance score $\langle W_{S_{max}}\rangle$, which has been used to rank the ortholog groups. Both values are shown normalized to the (0,1) range. The right ancillary columns indicate the two primary functional categories for the COGs assigned to each ortholog group, the description and identifier of which is shown adjacent. Highlighted descriptions denote ortholog groups also present in Fig 5.

Another bioinformatics analysis was performed with more diverse representation across the Alphaproteobacteria. This analysis included one representative from all of the Alphaproteobacteria orders with available complete or high-quality whole genome shotgun assemblies. Two orders (Magnetococcales & Holosporales) do not have GcrA, and the Minwuiales present a GcrA homolog but did not have any high-quality assemblies that could be used for this analysis. We identified 909 ortholog groups with orthologs in at least 11 of the 13 Alphaproteobacteria species and presenting one extended GANTC motif instance in at least one species were analyzed (S6 Data). Ortholog groups were sorted using the same criteria as in the *Caulobacteraceae/Hyphomonadaceae* analysis and their rank correlation with the *B*. *subvibrioides* GcrA ChIP-Seq dataset (ρ = 0.09, P<0.001) was analyzed. As earlier, a list containing the top 50 highest ranking genes was generated (Fig 6). Among the 13 species analyzed, *Candidatus Pelagibacter ubique* was found to have the least number of conserved orthologs (Fig 6). Even among the conserved orthologs, very few of them presented high probability of regulation by GcrA. For the remaining 12 species, even though the orthologs were conserved in most of them, the posterior probability of regulation for these orthologs varied significantly.

CcrM was found to be conserved in all 13 species, and with relatively high posterior probability of being regulated by GcrA in many of them, suggesting it is a core gene under the regulation of GcrA (Fig 6). However, this regulation seems to be lost in Caulobacterales (*Brevundimonas* and its close relatives specifically the freshwater genera) and its sister group Parvularculales (Figs 5 and 6). Another gene, a TetR/AcrR family regulator (COG 1309), was found to be conserved in 12 of the 13 species (absent in Pelagibacterales), with relatively high posterior probability of being regulated by GcrA in many of them, suggesting that it might also be part of the core GcrA regulon. Regulation of this gene by GcrA seems to be lost in Rhodospirillales and Parvularculales. As expected, the essential cell division gene *ftsZ* was found in all the 13 species and likely to be regulated by GcrA in some of them. CtrA seems to be an auxiliary gene in the GcrA regulon, as orthologs were found in all the species except in *Ca*. *P*. *ubique*, but only regulated by GcrA in Caulobacterales and Sphingomonadales. Interestingly, given the distance between the Caulobacterales and Sphingomonadales, this suggests that different rewiring events led to the uptake of *ctrA* regulation in these clades. Similarly, the DivL protein, which is involved in CtrA regulation, was found in all the species except in for *Ca*. *P*. *ubique* but likely to be regulated by GcrA only in few of them (including Caulobacterales), suggesting it is another auxiliary gene. Another gene, *popZ*, was found in 11 of the 13 species but likely to be regulated by GcrA only in the Rhizobiales, indicating a rewiring event. All in all, these results suggest that genes involved in DNA replication, cell division and *ctrA* regulation are conserved and regulated by GcrA within the order Caulobacterales, but that their conservation and regulation are not universal outside this order. This suggests that transcriptional rewiring of the GcrA regulon extends beyond the Caulobacterales and across the Alphaproteobacteria.

## Discussion

Given the fact that the two bacteria in this study are closely related evolutionarily, have the same dimorphic developmental life cycle, and live in the same freshwater environments [25], it was surprising to find such distinct regulons for critical regulatory systems. This does not appear to be a case where the regulator itself has mutated to recognize a different binding site, as MEME analysis of the *B*. *subvibrioides* data reveals the same basic GcrA binding site as in *C*. *crescentus*. While there has been some loss of gene content, horizontal gene transfer does not appear to be the major driver of regulon divergence. Of the 204 direct regulatory targets for *C*. *crescentus* GcrA, 147 orthologs are present in the *B*. *subvibrioides* genome. However, only 48 orthologs have GcrA binding sites while the remaining 99 orthologs have lost GcrA binding sites. The gain/loss of regulator binding sites for orthologs in different organisms is defined as transcriptional rewiring and appears to be the major driver for divergence between these regulons. Additionally, out of those 48 orthologs, 36 of them have GcrA binding sites but the change in transcription in a *gcrA* mutant did not meet the statistical cutoff used in the published *C*. *crescentus* study. Therefore, even though these genes have GcrA-binding sites, the actual *in vivo* effect of GcrA regulation may be minimal on those genes. This suggests that the number of common functional regulatory targets of GcrA in these two bacterial species is shockingly low given the similarities and relationship between them.

The level of transcriptional rewiring seen here is in sharp contrast to that observed for the AraC regulon of *E*. *coli* and *S*. *enterica* (both belong to the same family *Enterobacteriaceae*), where there was limited transcriptional rewiring [2]. Both *E*. *coli* and *S*. *enterica* belong to the same *Enterobacteriaceae* family and their average estimated divergence time is around 106 MYA, which is comparable to that of *C*. *crescentus* and *B*. *subvibrioides* who also belong to the same family (*Caulobacteraceae*) with average divergence time estimated around 155 MYA [39]. In addition, even though some transcriptional rewiring was found when FNR regulons were compared between the closely related Alphaproteobacteria *R*. *sphaeroides* and *R*. *capsulatus* [3], it was not as extensive as the rewiring seen here for GcrA. The bioinformatic analyses presented here suggest that there is only limited conservation of GcrA regulatory targets within the Caulobacterales, and that more extensive rewiring has taken place at the class level, with only the GcrA-CcrM connection being consistently preserved as a fundamental element. These data suggest that the GcrA/CcrM system may be more prone to transcriptional rewiring than other regulatory systems. If so, it is not clear why. Is it simply a function of size? The GcrA regulon is much larger than previously analyzed regulons; it may be that larger regulons simply demonstrate more variability between organisms. Perhaps it is a result of cellular function. Previously analyzed regulons were typically involved in specific metabolic pathways while GcrA is involved in cell cycle control; it may be that cell cycle control is more prone to rewiring because it is a global process that is used to coordinate multiple activities and each organism has its own unique suite of activities to control. One possibility may be simple probability. GcrA regulation appears to be largely dependent on the presence of a CcrM methylation site (GANTC), which is only a 5 bp sequence. Comparatively, this is much smaller and simpler than other regulator binding sites. Therefore, the probability of a methylation site being created or destroyed through random mutation would be much higher and occur much faster than other regulator binding sites, making the regulon more evolutionary labile.

However, can simple gain or loss of binding sites explain the data presented here? Thus far, the presence of a methylation site appears to be a major determining factor for regulation of a gene by GcrA, but is it the only thing? This study (as well as previous studies) shows that there are many thousands of methylation sites not bound by GcrA, and there are some genes regulated by GcrA that do not have methylation sites. In addition, 36 *B*. *subvibrioides* orthologs to *C*. *crescentus* GcrA targets still have GcrA binding peaks, but the change in expression in the *gcrA* mutant does not meet necessary cutoffs, suggesting there are other factors impacting expression of those genes. In those cases, transcriptional rewiring appears to have occurred in a non-binding-site specific way. One possibility that has yet to be investigated is that of GcrA effectors. There may be other biological molecules (e.g. proteins, small RNAs) that affect GcrA activity, and the gain/loss/misregulation of those could lead to, or appear as, major rewiring events. However, thus far there is no evidence for such molecules. Transcriptional rewiring seen in this system could be the combined result of multiple factors, including gain/loss of methylation sites as well as other unknown effectors. Without knowing what specifically determines a GcrA regulatory target, it is difficult to speculate why this system appears so prone to transcriptional rewiring.

The first indication that the GcrA/CcrM system differed between these two organisms was the finding that *ccrM* is non-essential in *B*. *subvibrioides* when grown in PYE medium [25] while it is essential in *C*. *crescentus* when grown in the same medium [20]. The differences in essentiality in PYE medium might be due to differences in gene expression of essential genes between the two organisms. Nine genes are categorized as essential and also show decreased expression in a *C*. *crescentus ccrM* mutant [23], but only 3 of those genes show similar results in *B*. *subvibrioides*. One potential target is the essential cell division gene *ftsN* which is significantly downregulated (>2-fold lower expression) in a *ccrM* mutant in *C*. *crescentus* but slightly upregulated (>1.56-fold higher expression) in a *B*. *subvibrioides ccrM* mutant. However, it is more likely that the explanation has to do with *ftsZ* expression and growth rate. One of the critical targets of CcrM regulation is *ftsZ*. Growth of *C*. *crescentus ccrM* mutants in PYE medium can be restored by exogenously expressing *ftsZ* [40]. It has also been shown that *C*. *crescentus ccrM* mutants can be cultured without exogenous *ftsZ* expression when the growth is slowed by using a minimal medium [40]. It is likely that slowing growth lengthens out the cell cycle, allowing FtsZ to accumulate to necessary levels despite having greatly decreased expression. While *C*. *crescentus* has a doubling time around 1.5 hrs in PYE medium, *B*. *subvibrioides* has a doubling time of around 6.5 hrs in the same growth medium [25]. Disruption of *ccrM* has no effect on the growth rate of *B*. *subvibrioides* in PYE likely because the organism grows slowly enough in that medium to permit sufficient FtsZ accumulation, even though *ftsZ* expression in that strain is reduced.

The slow growth rate of *B*. *subvibrioides* in PYE may explain the difference in *ccrM* essentiality, but why does *B*. *subvibrioides* grow so much slower than *C*. *crescentus* in the same growth media? The data generated in this study may suggest a hypothesis. In the *C*. *crescentus* predivisional cell stage, chromosome replication is initiated by DnaA which also induces production of GcrA. GcrA regulates many genes involved in chromosome replication as well as initiating cell division, initiating some polar structure biogenesis (pilus and flagellum), and inducing production of CtrA. CtrA completes cell division, completes several polar structure synthesis regimes, and represses further chromosome replication. The data here suggests that many of the polar structure biogenesis genes regulated in *C*. *crescentus* have been transcriptionally rewired. Most of the genes belonging to pilus biosynthesis that are regulated by GcrA in *C*. *crescentus* are not regulated by GcrA in *B*. *subvibrioides*, including *cpaB* (>1.17 fold lower expression, P>0.081), *cpaD* (>1.07 fold lower, P>0.6), *cpaE* (>1.003-fold lower, P>0.93), and *cpaF* (>1.01 fold lower expression, P>0.79). Similarly, flagellar genes *flhB*, *pflI*, *fliX*, *fliR*, *fliQ*, *fliM*, that are regulatory targets of GcrA in *C*. *crescentus* are not regulated by GcrA in *B*. *subvibrioides*. Polar development genes *popZ* and *podJ*, which are regulated by GcrA in *C*. *crescentus*, were not regulated by GcrA in *B*. *subvibrioides*. In the case of *podJ*, even though there was a GcrA peak in its promoter, RNA-seq showed >1.44-fold higher expression in *gcrA* mutant compared to WT. For *popZ*, RNA-seq showed only >1.13-fold lower expression in *gcrA* mutant compared to WT and no GcrA peak was found in the promoter region. Furthermore, none of the genes involved in holdfast biosynthesis were found to be regulated by GcrA in *B*. *subvibrioides* either due to lack of a GcrA peak in the promoter, because they did not meet the cut off of P<0.01, or both. These structures are still clearly made in *B*. *subvibrioides* [25], but the timing and regulation of their synthesis is now in question. If *B*. *subvibrioides* rewired some of the processes usually under the control of GcrA to a later regulator, it may be less able to compress its cell cycle into a smaller time frame when nutrients are abundant, which manifests as a different growth rate in the same medium.

While the regulation of many flagellum biosynthesis targets was not conserved between organisms, three genes involved in flagellum positioning were found to be common to both GcrA regulons. Those genes are *flbA*, *tipF* and *dgcA*. In *C*. *crescentus* (and possibly in *B*. *subvibrioides*), *tipF* is a cell cycle regulated gene which is expressed by GcrA in the early predivisional stage [30]. The main function of TipF is to select the flagellum assembly site in the early predivisional stage [31]. TipF localizes to the pole opposite to the stalk [30], and recruits PflI (and later other proteins such as FliF, FliG and FliM) which is required for flagellum positioning [31]. The integration of the flagellum positioning system into the GcrA regulon ensures the positioning system is active prior to flagellum biosynthesis initiated by CtrA. TipF has a C-terminal degenerate EAL domain which can bind to, but not degrade c-di-GMP [30]. In *C*. *crescentus*, TipF levels mirror c-di-GMP levels, and binding of c-di-GMP to TipF activates its recruitment of other flagellum positioning proteins [31]. The c-di-GMP synthesizing gene *dgcA* is also conserved in both regulons. The *dgcA* gene is cell cycle regulated in *C*. *crescentus*, and its expression pattern matches that of *tipF*. It is interesting to note that GcrA regulates *dgcA* but no other c-di-GMP metabolizing enzymes, including the more well-known *C*. *crescentus* enzymes *dgcB*, *pdeA* or *pleD*. It is tempting to speculate that the co-regulation of *tipF* and *dgcA* by GcrA in both organisms indicates they have a functional relationship in the cell. Perhaps the regulation of these genes by GcrA may offer an avenue into the exploration of their function.

One caveat of this study is the fact that the GcrA regulon in *C*. *crescentus* was identified using synchronized cells [19] whereas mixed cell populations of *B*. *subvibrioides* were used because there is no synchronizable strain of *B*. *subvibrioides*. However, re-analysis of the data using relaxed cutoffs did not significantly improve the common set of GcrA-regulated genes between both organisms. Additionally, a different study (Holtzendorff *et al*. (2004)) using mixed cell populations of *C*. *crescentus* and microarrays found 125 genes that were misregulated in *gcrA* compared to WT (P<0.05) [11]. When those 125 misregulated genes from Holtzendorff *et al*. (2004) (unsynchronized *C*. *crescentus* cells) were compared to the misregulated genes from Haakonsen *et al*. (2015) (synchronized *C*. *crescentus* cells) study, 80 genes were in common. When those 125 genes from Holtzendorff *et al*. (2004) were compared with the 131 genes identified in this study only 5 genes were common between both these datasets (Table K in S1 Text). This suggests that the differences in approach did not have a significant impact on regulon comparison and increases the validity of the conclusions of this study.

The model proposed by Haakonsen *et al*. (2015) suggests that GcrA interacts with the housekeeping sigma factor (σ^70^) in the RNA polymerase holoenzyme first and then is recruited to promoters. However, it was also proposed GcrA does not activate all the promoters it binds to, only those that have methylated promoters with the extended motif of YGAKTCG. The RNA-seq and ChIP-seq data from *B*. *subvibrioides* reported here suggests some small but notable disagreements with the *C*. *crescentus* model. ChIP-seq data showed that *B*. *subvibrioides* GcrA bound to intergenic regions of several hundred different genes, but only increased transcription of a small subset of those genes. However, MEME analysis of those promoters did not result in the detection of an extended motif like in *C*. *crescentus*, just the basic CcrM methylation motif with a small preference for C before the G. If this is true, it is not clear how GcrA distinguishes between promoters that it activates and those it just binds to without activation. Also, some genes were misregulated in *gcrA* mutants and a GcrA peak was also detected in their respective promoter regions, but no methylation site was found in those GcrA peaks. This suggests that GcrA is able to regulate expression of a small number of genes in a methylation-independent manner. This deviates from the proposed *C*. *crescentus* model, though it should be noted that GcrA binding to sequences that do not have methylated GANTC sites has been reported in *C*. *crescentus* as well [18,19].

The data presented in this study suggest that despite being closely related and living in the same habitat, CcrM methylation and GcrA regulate surprisingly different genes in *C*. *crescentus* and *B*. *subvibrioides*. Genes involved in DNA replication, cell division, and regulation of CtrA were common regulatory targets in both these organisms, and bioinformatics analysis suggests these may be common targets in the larger Alphaproteobacteria group, though conservation outside the Caulobacterales is more variable. Further testing in different and more varied organisms is needed to determine how the GcrA/CcrM system is customized to each organism and its own particular physiology.

## Materials and methods

### Bacterial strains and growth conditions

All strains and plasmids used in this study are listed in Table A in S1 Text. *B*. *subvibrioides* Δ*gcrA* and *ccrM*::pNPTS139 were previously constructed [25]. All *B*. *subvibrioides* strains were grown in PYE medium (2 g l^−1^ peptone, 1 g l^−1^ yeast extract, 0.3 g l^−1^ MgSO_4_.7H_2_O, 0.0735 g l^−1^ CaCl_2_.2H_2_O) at 30°C. Kanamycin was supplemented at 20 μg ml^-1^ and tetracycline was supplemented at 2 μg ml^-1^ when necessary. *Escherichia coli* strains were grown in LB media (10 g l^−1^ tryptone, 5 g l^−1^ yeast extract, 10 g l^−1^ NaCl) at 37°C. Kanamycin was supplemented at 50 μg ml^−1^ and tetracycline was supplemented at 12 μg ml^-1^ when necessary.

### Strain construction

For GcrA purification, the coding region of *gcrA* was amplified using primers GcrAhisF and GcrAhisR (see Table B in S1 Text), digested using NdeI and EcoRI, and cloned into pET28a (Millipore) to create pSA100, which created a construct where GcrA was given a N-terminal 6X his-tag. This plasmid was introduced into *E*. *coli* Bl21 (DE3) by electroporation.

The plasmid for replacing the CtrA binding site in *sciP* promoter (Bresu_1445) was constructed by amplification of two fragments. The first fragment was amplified using Upbresu1445F and Upbresu1445R (see Table B in S1 Text). The reverse primer (Upbresu1445R) was synthesized in such a way that one of CtrA half binding site (TAAA) was replaced to GGCC. The second fragment was amplified using primers Dnbresu1445F and Dnbresu1445R). The forward primer (Dnbresu1445F) was synthesized with another CtrA half site (TTAG) replaced to GGCC. Both these fragments were cloned into pNPTS138 (M.R.K. Alley, unpublished) using Gibson Assembly (New England Biolabs) to produce pSA400. The end result was a construct where both half-sites of the CtrA binding site were mutated, centered in ~1500 bp of otherwise homologous sequence. This plasmid was electroporated into WT and *gcrA* strains and plated into PYE + kanamycin plates. Kanamycin resistance colonies were grown in the absence of selection, then plated on PYE plates containing 3% sucrose. Sucrose resistant colonies were screened for the replacement of the *ctrA* binding site in *sciP* promoter by DNA sequencing using primers Conf1445F and Conf1445R. This resulted in two strains: P_sciP-no ctrA_ and *gcrA* + P_sciP-no ctrA_.

### Single molecule real time (SMRT) sequencing

Genomic DNA was extracted from exponentially growing wild-type and *ccrM*::pNPTS139 *B*. *subvibrioides* strains once using the DNeasy Blood & Tissue Kit (Qiagen) as described in the manual. The concentration of genomic DNA was measured using a Thermo Nanodrop 2000 (Thermo Scientific). The samples were then sent for SMRTbell library preparation followed by sequencing using a Pacbio RS II instrument at the Arizona Genomics Institute, University of Arizona. De novo assembly was performed using BLASR. SMRT Portal was used for data analysis. For motif analysis, a default Quality Value (QV) (defined as an estimate for accuracy of basecall during sequencing) of 30 was used which corresponds to 99.9% accuracy. To identify adenine methylation, Interpulse duration (IPD) ratio was used. IPD is a time duration for a polymerase to incorporate successive nucleotides. If there is a presence of a methylated base during incorporation, then the IPD value increases compared to a control that lacks methylated base (in silico control) at the same site. IPD ratio <1 was treated as unmethylated adenine and IPD ratio > 1 was treated as methylated adenine.

### RNA sequencing and data analysis

Total RNA from mid log stage cells was extracted using Max Bacterial Enhancement Reagent (Ambion) with TRIzol reagent (Ambion) and PureLink RNA Mini Kit (Invitrogen). All RNA samples were extracted from cultures grown independently in triplicates. The concentration was measured using a Nanodrop 2000 and sent for sequencing at the Center for Genomics and Bioinformatics at Indiana University, Bloomington. RNA integrity was assessed by an Agilent 2100 Bioanalyzer (Agilent Technologies). Messenger RNA enrichment was done by removing rRNA using MICROBExpress rRNA removal kit (Ambion). Before library preparation, cDNA was synthesized complementary to mRNA using random primers and Reverse Transcriptase. Second strands complementary to newly synthesized strands were synthesized, creating a double stranded DNA from the mRNA template. This DNA was used for library preparation using Nextera XT DNA Library Prep Kit (Illumina) followed by Illumina sequencing and analysis. After sequencing, raw reads were viewed by FASTQC, followed by adapter trimming and quality clipping by Trimmomatic and low-quality reads were discarded. Good quality reads were mapped to *B*. *subvibrioides* genome using Bowtie2. Differential gene expression analysis was performed using DEseq2 package.

### GcrA purification and antibody production

GcrA expression, cell lysis and purification were performed by following the QIAexpressionist manual (Qiagen). Briefly, 500 ml of culture was grown to mid log stage and GcrA production was induced by addition of IPTG to a final concentration of 0.4 mM. Cells were induced for 4–5 hours at 37°C (200 rpm), collected by centrifugation (4000 x g, 20 mins, 4°C), and cells were resuspended in lysis buffer (50 mM NaH_2_PO_4_, 300 mM NaCl, 10 mM imidazole, pH 8.0). Lysozyme (Thermo Scientific) was added to a final concentration of 1 mg/ml and the solution was incubated on ice for 30 min followed by sonication (amplitude 50%, total duration 2 mins with 30s cooling time) and centrifugation (10000 x g, 20 mins, 4°C). The supernatant was collected, 1 ml of 50% Ni-NTA slurry was added to 4 ml of cleared lysate and mixed gently for 1 hour at 4°C, followed by loading into a column. The column was washed with 4 ml of wash buffer (50 mM NaH_2_PO_4_, 300 mM NaCl, 20 mM imidazole, pH 8.0) twice and the protein was eluted four times using 0.5 ml elution buffer (50 mM NaH_2_PO_4_, 300 mM NaCl, 250 mM imidazole, pH 8.0). The protein size was verified by SDS PAGE. To further purify GcrA, gel filtration was utilized. Chromatography resin (Superdex 75 Prep Grade, GE) was pre-equilibrated with running/storage buffer (50 mM Tris-HCl (pH 8.5), 200 mM NaCl, 5% glycerol) in a 30 cm column. GcrA containing protein fractions were added on the top of the column and eluted with running buffer (50 mM Tris-HCl (pH 8.5), 200 mM NaCl, 5% glycerol). Eluted samples were collected and verified by SDS PAGE. GcrA was concentrated using 10 kDa Centrifugal Filter Units (Micron-10, Millipore). The concentration of GcrA was measured by Pierce BCA protein assay kit (Thermo scientific). Purified GcrA was used to produce rabbit anti-GcrA polyclonal antibodies (Thermo Fisher). The specificity of antibody was verified by Western blot where a single band of correct size (~ 18kDa) was detected in whole cell lysate of WT and absent in *gcrA* mutant strain.

### Chromatin Immunoprecipitation (ChIP) sequencing and data analysis

ChIP was performed as previously described [18,19]. Wild-type cells were grown in triplicates to mid-log stage and molecular crosslinking was performed by adding 10 mM sodium phosphate (pH 7.6) and 1% formaldehyde at room temperature for 10 min, followed by incubation on ice for 30 min. Crosslinking was stopped by adding glycine to final concentration of 100 mM and incubated for 5 mins at room temperature followed by 15 min on ice. Cells were centrifuged at 5000 x g at 4°C for 5 mins. The supernatant was removed, and cells were resuspended in 1 ml of 1X phosphate buffered saline (PBS, pH 7.4). This step was repeated 2 more time and cells were finally resuspended in 500 μL of TES buffer (10 mM Tris-HCl (pH 7.5), 1 mM EDTA, 100 mM NaCl) to which 2 μL of 20,000 U/μL lysozyme was added and the solution was then incubated for 15 min at room temperature. ChIP buffer (16.7 mM Tris-HCl (pH 8.1), 167 mM NaCl, 1.1% Triton X-100, 1.2 mM EDTA) containing Protease inhibitors (Roche cOmplete EDTA-free tablets) solution (prepared as per manufacture’s instruction) was prepared and 500 μL was added. After incubating for 10 mins at 37°C, the lysates were sonicated on ice to generate DNA fragments of 0.3–0.5 kbp (assessed by agarose gel electrophoresis) followed by centrifugation at 14000 x g for 5 mins at 4°C. Supernatant was collected and the protein concentration in the supernatant was measured by Pierce BCA protein assay kit (Thermo Scientific). A protein solution containing 500 μg was diluted to a final volume of 1 mL using ChIP buffer (containing protease inhibitor) with 0.01% SDS, and pre-cleared with 80 μL of Protein-A agarose (Invitrogen) (pre-blocked with 100 μg bovine serum albumin (BSA) overnight) for 1 hr at 4°C in a shaking platform. After centrifugation (3000 x g, 1 min), supernatant was collected and 10% of the supernatant was stored at -80 °C and used as total chromatin input DNA. Anti-GcrA sera (1:500 dilution) was added to the remaining supernatant with 80 μl of Protein-A agarose (Invitrogen) (pre-blocked with 100 μg BSA overnight) and incubated at 4°C overnight. The pellet was washed with low salt wash buffer (0.1% SDS, 1% Triton X-100, 2 mM EDTA, 20 mM Tris-HCl (pH 8.1), 150 mM NaCl) followed by centrifugation (5000 x g, 2 mins) at 4°C and the supernatant was discarded. This washing step was repeated with high salt wash buffer (0.1% SDS, 1% Triton X-100, 2 mM EDTA, 20 mM Tris-HCl (pH 8.1), 500 mM NaCl), LiCl wash buffer (0.25 M LiCl, 1% NP-40, 1% deoxycholate, 1 mM EDTA, 10 mM Tris-HCl (pH 8.1)) and finally twice with TE buffer (10 mM Tris-HCl (pH 8.1),1 mM EDTA). Elution was performed twice from the beads with 250 μL of freshly prepared elution buffer (1% SDS, 0.1 M NaHCO_3_) followed by addition of NaCl to a final concentration of 300 mM as well as 2 μl of RNase A (10mg/ml) (Thermo scientific). Reverse crosslinking was done overnight by incubating at 65 °C. Samples were then incubated at 45 °C for 2 hr with 5 μL of Proteinase-K (20 mg/ml) in the presence of 40 mM EDTA (pH 8.0) and 40 mM Tris-HCl (pH 6.8). Phenol:chloroform:isoamyl alcohol (25,24:1) was used for DNA extraction which was followed by addition of 1/10 volume of 3M sodium acetate (pH 5.2), 100 μg glycogen and 1 volume of cold isopropanol. The solution was stored at -20°C overnight. Next day, centrifugation (16000 x g, 30 min) was done to pellet glycogen containing DNA and washed with 75% ethanol followed by centrifugation (16000 x g, 2 min) twice and finally resuspended in 100 μl of TE buffer (pH 8.0). Enrichment of DNA was verified by qPCR and sent for Illumina sequencing at The Biodesign Institute, Arizona State University.

The raw Illumina 2x75bp pair-end reads were quality checked using FastQC v0.10.1, followed by adapter trimming and quality clipping by Trimmomatic 0.35. Any reads with start, end or the average quality within 4 bp windows falling below quality scores 18 were trimmed. The clean reads were aligned to the reference genome *Brevundimonas subvibrioides* ATCC 15264 by Bowtie2 version 2.2.9. Library insert size was checked by Picard Tool (https://broadinstitute.github.io/picard/). Library complexity was checked by NRF (nonredundancy fraction), defined as the number of unique start positions of uniquely mappable reads divided by number of uniquely mappable reads. IGVtools and bamCompare from deepTools were employed for comparing two BAM files based on the number of mapped reads. First the genome is partitioned into bins of equal size and then the number of reads in each bin is counted. The log2 value for the ratio of number of reads per bin of each sample was reported for IGV visualization and compared between each pair. With 95% correlation, three biological replicates were combined for peak identification. MACS2 was used for peaks calling with 0.05 FDR cutoff.

### RT-qPCR

Total RNA from strains WT, WT P_sciP-no ctrA_, *gcrA*, and *gcrA* P_sciP-no ctrA_ grown to mid log stage was extracted using Max Bacterial Enhancement Reagent (Ambion) with TRIzol reagent (Ambion) and PureLink RNA Mini Kit (Invitrogen). All RNA samples were extracted from cultures grown independently in triplicates. RNA concentration was measured, and equal amount of total RNA was treated with 10 μl DNase I (Thermo) for 30 min at 37°C. DNase was inactivated by addition of EDTA and incubation at 65°C for 10 min. cDNA synthesis was done using Quantitect Reverse Transcription Kit (Qiagen). Real time PCR was performed using SciP primers (qpcrsciPF and qpcrsciPR, see Table B in S1 Text for sequences) in a Rotor Gene Q (Qiagen) using Quantitect SYBR Green kit (Qiagen). The Ct values were normalized using reference gene (Bresu_2921refF and Bresu_2921refR primers) and 2^−ΔΔCT^ method was used for calculation of relative *sciP* expression level.

For validation of RNA seq data, expression levels of 10 genes that showed differential expression in the *ccrM* dataset (S1 Fig (Bottom)) were analyzed by RT-qPCR using RNA extraction, cDNA synthesis, and data analysis as described above. Another set of 10 genes that showed differential expression in the *gcrA* dataset were also analyzed in the same fashion (S1 Fig (Top)).

### Bioinformatics methods

Comparative genomics analyses were performed with CGB, a bioinformatics pipeline that integrates all the necessary steps for assessing the conservation of regulatory sites upstream of orthologs [41,42]. Given one or more collections of known binding sites for a transcription factor, CGB downloads target genomes, predicts operons and scans the upstream regions of operon lead genes for transcription factor-binding site instances. It then predicts orthologs across all analyzed genomes and infers the posterior probability of regulation based on the presence of transcription factor-binding sites upstream of each operon. Genome sequences for all the Alphaproteobacteria species analyzed here were obtained from the NCBI RefSeq database. Extended GANTC motifs were obtained for *C*. *crescentus* [19] and *B*. *subvibrioides* (this work), and combined as a mixture model to approximate the extended GANTC motif in target Alphaproteobacteria species. Extended GANTC motif instances were considered statistically significant when the PSSM score threshold satisfied the equality between the negative logarithm of the false positive rate (FPR) and the information content (IC) of the motif [43]. Spearman rank correlations and permutation tests to assess the correlation between average GANTC site scores and ChIP-seq enrichment were performed with custom Python scripts.

### Accession numbers

RNA-seq and ChIP-seq data have been deposited at the Gene Expression Omnibus (GEO) under accession numbers GSE138844 (RNA-seq) and GSE138845 (ChIP-seq).

## Supporting information

S1 Data List of all the motifs detected by SMRT sequencing in WT and *ccrM* mutant. For GANTC motifs, the fully methylated, hemi-methylated and unmethylated sites along with their IPD ratio are also shown.
(XLSX)

S2 Data List of genes that were found misregulated in *ccrM* mutant compared to WT (P<0.01 and >2-fold). (XLSX)

S3 Data List of genes that were found misregulated in *gcrA* mutant compared to WT (P<0.01 and >2-fold). (XLSX)

S4 Data List of total GcrA peaks detected by ChIP-seq. (XLSX)

S5 Data Bioinformatics analysis for GcrA regulon across the order Caulobacterales. (XLSX)

S6 Data Bioinformatics analysis for GcrA regulon across Alphaproteobacteria. (XLSX)

S1 Text List of strains, plasmids, primers used in the study. This is file also includes other data discussed in the main text.
(DOCX)

S1 Fig Validation of RNA seq data with RT-qPCR. In order to confirm our RNA-seq data, 10 genes each that were misexpressed in *gcrA* mutant (top) or *ccrM* mutant (bottom) were taken and RT-qPCR was performed in triplicates which are consisted in direction and amplitude except for Bresu_1213 (*ctrA*) in *ccrM*. Blue bars show log fold change expression obtained from RT-qPCR and red bars show log fold change expression obtained from RNA-seq data, error bars show standard deviation. The expression of two genes: Bresu_1037 and Bresu_2926 did not change in *gcrA* or *ccrM* compared to WT in both RNA-seq and RT-qPCR and were taken as reference genes.
(TIFF)

S2 Fig GcrA binding sites in the *B*. *subvibrioides sciP* promoter region are not sufficient for transcription. A) Comparison of *sciP* promoter regions between *C*. *crescentus* and B. *subvibrioides*. In *C*. *crescentus*, *sciP* is expressed by the binding of CtrA which is located in the promoter region. In *B*. *subvibrioides*, in addition to the CtrA binding sites, two GANTC methylation sites (shown by asterisk) are present (located at -71bp and +144bp from the start codon) and ChIP-seq data shows that GcrA binds to the GANTC site located at -71bp form start codon (shown in histograms). B) GcrA does not appear to be involved in activation of *sciP* in *B*. *subvibrioides*. The CtrA binding site of in the *sciP* promoter was mutated to GGCC-N7-GGCC (P_*sciP-no ctrA*_), in the wild-type and *gcrA* strains. RT-qPCR was performed (in triplicates, error bars show standard deviation) to quantify expression, with expression levels normalized to wild-type. Mutation of the CtrA binding site caused a dramatic loss of *sciP* expression, indicating that GcrA alone is not sufficient to induce *sciP*.
(TIFF)
