# Supplementary material for: Transcriptional rewiring of the GcrA/CcrM bacterial epigenetic regulatory system in closely related bacteria
Source: PLoS Genet. 2021 Mar 11;17(3):e1009433. doi: 10.1371/journal.pgen.1009433 (PMC7987155; doi:10.1371/journal.pgen.1009433)
Supplement: S1 Text — This is file also includes other data discussed in the main text. (DOCX) [file pgen.1009433.s007.docx]

**Table A: List of strains and plasmids used in this study**

| **Strain** | **Characteristics** | **Source** |
| --- | --- | --- |
| DH5alpha | General cloning strain | Bioline |
| BL21 λDE3 | Strain for protein expression and purification | Novagen |
| PC0217 | pSA100 in BL21 λDE3 | This study |
| PC0273 | pSA400 in DH5alpha | This study |
| *B. subvibroides* |  |  |
| ATCC15264 | Wild-type | ATCC |
| PC0008 | ATCC15264 Δ*gcrA* | Curtis & Brun (2014) |
| PC0021 | ATCC15264 *ccrM*::pNPTS139 | Curtis & Brun (2014) |
| PC0274 | pSA400 in WT to create WT P_sciP-no ctrA_ | This study |
| PC0275 | pSA400 in Δ*gcrA* to create Δ*gcrA* P_sciP-no ctrA_ | This study |
| **Plasmids** | **Characteristics** | **Source** |
| pNPTS138 | Kan^R^, Suc^S^ | M.R.K. Alley, unpublished |
| pET28a | Expression vector with 6X-his tag | Millipore |
| plac290 | lacZ transcriptional fusion vector for ß-galactosidase assay | Gober and Shapiro (1992) |
| pSA100 | pET28a-GcrA-6Xhis tag | This study |
| pSA400 | pNPTS138+ upbresu1445+dnbresu1445for replacing *ctrA* binding site in *sciP* promoter | This study |

**Table B: Oligonucleotides used in this study**

| Primer | Sequence |
| --- | --- |
| GcrAhisF | CGACATATGACCGCAGGCTGGACCGAAG |
| GcrAhisR | GCCGAATTCCTAGATGTAACGCCGCAGG |
| Upbresu1445F | ATTGAAGCCGGCTGGCGCCAAGCTTACGGCACGACGCCGTCCAACGGC |
| Upbresu1445R | GCCGGGCCGGGGCCGCGTCGTGGTGAAGGAATGGATAAAATCC |
| Dnbresu1445F | CGACGCGGCCCCGGCCCGGCCCGGCGCTTGGCTACAGTCACTGTC |
| Dnbresu1445R | CGTCACGGCCGAAGCTAGCGAATTCGACGGCGTCGCGGAAGCGGCTTTCG |
| RTPCRsciPF | CGCTGGGTGATCCGTCGCAAG |
| RTPCRsciPR | GTTCTGGATGCGCGTGGTGCG |
| Bresu_2921refF | CCGACCTGACGGGTTTGGAAGG |
| Bresu_2921refR | CGCCAAGGATGACCCGGTGAG |
| Conf1445F | GCTGATCGCCAAGTAACGCAGCG |
| Conf1445R | GGGACGATTTCGAGGCCGGAC |

**Table C: List of downregulated genes with at least one GANTC site in their promoter in *ccrM* mutant compared to WT in *B. subvibrioides***

| **New Locus Tag** | **Old Locus Tag** | **Description** | **GANTC sites** | **Distance from nearest site (up to 200 bp)** | **Part of operon** | **First gene of operon** |
| --- | --- | --- | --- | --- | --- | --- |
|  |  |  |  |  |  |  |
| BRESU_RS01200 | Bresu_0240 | response regulator | 1 | 130 | Yes | Yes |
| BRESU_RS01205 | Bresu_0241 | hypothetical protein | 1 | 130 | Yes | BRESU_RS01200 |
| BRESU_RS03340 | Bresu_0668 | hypothetical protein | 1 | 16 | No |  |
| BRESU_RS04200 | Bresu_0843 | hybrid sensor histidine kinase/response regulator | 2 | 47 | No |  |
| BRESU_RS04250 | Bresu_0853 | flagellar motor protein MotB | 1 | 157 | No |  |
| BRESU_RS04745 | Bresu_0953 | extensin | 1 | 15 | No |  |
| BRESU_RS04825 | Bresu_0969 | cell wall hydrolase | 2 | 154 | No |  |
| BRESU_RS05470 | Bresu_1100 | HNH endonuclease | 1 | 85 | No |  |
| BRESU_RS06525 | Bresu_1318 | hypothetical protein | 2 | 30 | Yes | BRESU_RS06530 |
| BRESU_RS06530 | Bresu_1319 | adenylosuccinate lyase | 2 | 30 | Yes | Yes |
| BRESU_RS06755 | Bresu_1366 | hypothetical protein | 1 | 39 | No |  |
| BRESU_RS08130 | Bresu_1647 | limonene-1,2-epoxide hydrolase | 1 | 39 | No |  |
| BRESU_RS08625 | Bresu_1743 | ClpP | 5 | 13 | No |  |
| BRESU_RS08815 | Bresu_1779 | spermidine synthase | 1 | 55 | No |  |
| BRESU_RS09235 | Bresu_1865 | DNA topoisomerase IV subunit B | 2 | 29 | Yes | BRESU_RS09240 |
| BRESU_RS09420 | Bresu_1901 | membrane protein | 1 | 53 | Yes | BRESU_RS09425 |
| BRESU_RS09865 | Bresu_1987 | hypothetical protein | 1 | 9 | No |  |
| BRESU_RS09945 | Bresu_2002 | hypothetical protein | 4 | 20 | Yes | BRESU_RS09950 |
| BRESU_RS09950 | Bresu_2003 | hypothetical protein | 4 | 20 | Yes | Yes |
| BRESU_RS10085 | Bresu_2033 | DNA-cytosine methyltransferase | 1 | 82 | Yes | BRESU_RS10090 |
| BRESU_RS10115 | Bresu_2039 | hypothetical protein | 2 | 83 | No |  |
| BRESU_RS11535 | Bresu_2323 | hypothetical protein | 1 | 125 | No |  |
| BRESU_RS11700 | Bresu_2357 | colicin V production protein CvpA | 2 | 45 | No |  |
| BRESU_RS11765 | Bresu_2369 | MFS transporter | 1 | 24 | Yes | Yes |
| BRESU_RS11830 | Bresu_2382 | DNA mismatch repair protein MutL | 1 | 104 | No |  |
| BRESU_RS12010 | Bresu_2416 | MarR family transcriptional regulator | 1 | 46 | No |  |
| BRESU_RS12240 | Bresu_2462 | amino acid permease | 1 | 33 | Yes | Yes |
| BRESU_RS12785 | Bresu_2568 | cytochrome b | 2 | 16 | Yes | Yes |
| BRESU_RS12795 | Bresu_2570 | hypothetical protein | 1 | 6 | No |  |
| BRESU_RS13080 | Bresu_2627 | class IV aminotransferase | 2 | 89 | Yes | BRESU_RS13085 |
| BRESU_RS13085 | Bresu_2628 | DNA gyrase subunit A | 2 | 89 | Yes | Yes |
| BRESU_RS14540 | Bresu_2914 | hypothetical protein | 1 | 42 | No |  |
| BRESU_RS14550 | Bresu_2918 | hypothetical protein | 1 | 145 | No |  |
| BRESU_RS14665 | Bresu_2934 | MFS transporter | 1 | 23 | No |  |
| BRESU_RS14750 | Bresu_2951 | dihydroxy-acid dehydratase | 1 | 184 | No |  |
| BRESU_RS14785 | Bresu_2958 | hypothetical protein | 3 | 10 | No |  |
| BRESU_RS14835 | Bresu_2968 | hypothetical protein | 2 | 7 | Yes | BRESU_RS14850 |
| BRESU_RS14950 | Bresu_2992 | flagellar hook-basal body protein FliE | 1 | 36 | Yes | BRESU_RS14960 |
| BRESU_RS14970 | Bresu_2996 | flagellar biosynthetic protein FliP | 1 | 140 | Yes | BRESU_RS14965 |
| BRESU_RS15075 | Bresu_3016 | CckA | 1 | 19 | No |  |
| BRESU_RS15155 | Bresu_3032 | DNA-3-methyladenine glycosylase | 1 | 11 | No |  |
| BRESU_RS15855 | Bresu_3174 | thiol reductase thioredoxin | 1 | 65 | No |  |

**Table D: List of upregulated genes with at least one GANTC site in their promoter in *ccrM* mutant compared to WT in *B. subvibrioides***

| **New Locus Tag** | **Old Locus Tag** | **Description** | **GANTC sites** | **Distance from nearest site (up to 200 bp)** | **Part of operon** | **First gene of operon** |
| --- | --- | --- | --- | --- | --- | --- |
| BRESU_RS00880 | Bresu_0175 | hypothetical protein | 2 | 3 | No |  |
| BRESU_RS01925 | Bresu_0383 | hypothetical protein | 2 | 139 | No |  |
| BRESU_RS05300 | Bresu_1065 | hypothetical protein | 2 | 53 | No |  |
| BRESU_RS06020 | Bresu_1213 | CtrA | 5 | 116 | No |  |
| BRESU_RS06915 | Bresu_1401 | integrase | 3 | 9 | No |  |
| BRESU_RS10365 | Bresu_2089 | transcriptional regulator, Crp/Fnr family | 2 | 45 | No |  |
| BRESU_RS12005 | Bresu_2415 | MFS transporter | 1 | 5 | No |  |
| BRESU_RS12015 | Bresu_2417 | CoA transferase | 1 | 143 | Yes | Yes |
| BRESU_RS12020 | Bresu_2418 | 2-hydroxyglutaryl-CoA dehydratase | 1 | 143 | Yes | BRESU_RS12015 |
| BRESU_RS13395 | Bresu_2692 | UDP-N-acetylmuramate--L-alanine ligase | 2 | 50 | Yes | Yes |
| BRESU_RS14700 | Bresu_2941 | LacI family transcriptional regulator | 1 | 112 | No |  |
| BRESU_RS15525 | Bresu_3108 | DEAD/DEAH box helicase | 2 | 108 | No |  |
| BRESU_RS16790 | Bresu_0931 | hypothetical protein | 1 | 30 | Yes | Yes |
| BRESU_RS16955 | Bresu_2559 | hypothetical protein | 2 | 29 | No |  |

**Table E: List of common genes under the regulation of CcrM in *C. crescentus* and *B. subvibrioides* (cut off P<0.01 and >2-fold in both organisms)**

| Gene name | Description | *C. crescentus* gene | GANTC sites | *B. subvibrioides* gene | GANTC sites |
| --- | --- | --- | --- | --- | --- |
| *parE* | DNA topoisomerase IV subunit B | CCNA_02052 | 2 | Bresu_1865 | 1 |
| *gyrA* | DNA gyrase subunit A | CCNA_01651 | 2 | Bresu_2628 | 2 |
| *thyA* | thymidylate synthase | CCNA_02208 | 1 | Bresu_2476 | 0 |
|  | Hypothetical protein | CCNA_02091 | 1 | Bresu_2656 | 0 |

**Table F: List of downregulated genes under the direct regulation of GcrA in *B. subvibrioides* (P<0.01, >2-fold and presence of GcrA peak in the promoter region (-100 bp to +100 bp of the translational start site))**

| **Locus Tag** | **Old Locus Tag** | **Description** | **GANTC sites** | **Distance** | **GcrA peak** | **Part of operon** | **First gene of operon** | **Notes** |
| --- | --- | --- | --- | --- | --- | --- | --- | --- |
| BRESU_RS04825 | Bresu_0969 | cell wall hydrolase | 1 | -45 | yes | no |  |  |
| BRESU_RS16670 | Bresu_0053 | peptidoglycan-binding protein | 0 |  | yes | no |  | 3 GANTC sites located upstream >200bp from start codon |
| BRESU_RS00280 | Bresu_0054 | hypothetical protein | 1 | -22 | yes | no |  |  |
| BRESU_RS00300 | Bresu_0058 | glycoside hydrolase | 0 |  | yes | yes | BRESU_RS00430 |  |
| BRESU_RS00305 | Bresu_0059 | hypothetical protein | 0 |  | yes | yes | BRESU_RS00430 |  |
| BRESU_RS00310 | Bresu_0060 | hypothetical protein | 0 |  | yes | yes | BRESU_RS00430 |  |
| BRESU_RS00315 | Bresu_0061 | hypothetical protein | 0 |  | yes | yes | BRESU_RS00430 |  |
| BRESU_RS00320 | Bresu_0062 | hypothetical protein | 0 |  | yes | yes | BRESU_RS00430 |  |
| BRESU_RS00325 | Bresu_0063 | hypothetical protein | 0 |  | yes | yes | BRESU_RS00430 |  |
| BRESU_RS00330 | Bresu_0064 | hypothetical protein | 0 |  | yes | yes | BRESU_RS00430 |  |
| BRESU_RS00335 | Bresu_0065 | hypothetical protein | 0 |  | yes | yes | BRESU_RS00430 |  |
| BRESU_RS00340 | Bresu_0066 | hypothetical protein | 0 |  | yes | yes | BRESU_RS00430 |  |
| BRESU_RS00345 | Bresu_0067 | hypothetical protein | 0 |  | yes | yes | BRESU_RS00430 |  |
| BRESU_RS00350 | Bresu_0068 | hypothetical protein | 0 |  | yes | yes | BRESU_RS00430 |  |
| BRESU_RS00355 | Bresu_0069 | hypothetical protein | 0 |  | yes | yes | BRESU_RS00430 |  |
| BRESU_RS00360 | Bresu_0070 | hypothetical protein | 0 |  | yes | yes | BRESU_RS00430 |  |
| BRESU_RS00365 | Bresu_0071 | phage tail length tape measure protein | 0 |  | yes | yes | BRESU_RS00430 |  |
| BRESU_RS16685 | Bresu_0073 | hypothetical protein | 0 |  | yes | yes | BRESU_RS00430 |  |
| BRESU_RS00380 | Bresu_0074 | hypothetical protein | 0 |  | yes | yes | BRESU_RS00430 |  |
| BRESU_RS00385 | Bresu_0075 | hypothetical protein | 0 |  | yes | yes | BRESU_RS00430 |  |
| BRESU_RS00390 | Bresu_0076 | hypothetical protein | 0 |  | yes | yes | BRESU_RS00430 |  |
| BRESU_RS00395 | Bresu_0077 | hypothetical protein | 0 |  | yes | yes | BRESU_RS00430 |  |
| BRESU_RS00400 | Bresu_0078 | ribonuclease G | 0 |  | yes | yes | BRESU_RS00430 |  |
| BRESU_RS00405 | Bresu_0079 | hypothetical protein | 0 |  | yes | yes | BRESU_RS00430 |  |
| BRESU_RS00410 | Bresu_0080 | peptidase | 0 |  | yes | yes | BRESU_RS00430 |  |
| BRESU_RS00415 | Bresu_0081 | phage portal protein | 0 |  | yes | yes | BRESU_RS00430 |  |
| BRESU_RS00420 | Bresu_0082 | hypothetical protein | 0 |  | yes | yes | BRESU_RS00430 |  |
| BRESU_RS00425 | Bresu_0083 | terminase | 0 |  | yes | yes | BRESU_RS00430 |  |
| BRESU_RS00430 | Bresu_0084 | hypothetical protein | 0 |  | yes | yes | yes | One GANTC site located 240bp up from start codon |
| BRESU_RS00440 | Bresu_0086 | hypothetical protein | 1 |  | yes | yes | BRESU_RS00470 |  |
| BRESU_RS00445 | Bresu_0087 | zinc finger CHC2-family protein | 1 |  | yes | yes | BRESU_RS00470 |  |
| BRESU_RS00450 | Bresu_0088 | hypothetical protein | 1 |  | yes | yes | BRESU_RS00470 |  |
| BRESU_RS00455 | Bresu_0089 | hypothetical protein | 1 |  | yes | yes | BRESU_RS00470 |  |
| BRESU_RS16690 | Bresu_0090 | hypothetical protein | 1 |  | yes | yes | BRESU_RS00470 |  |
| BRESU_RS00465 | Bresu_0091 | hypothetical protein | 1 |  | yes | yes | BRESU_RS00470 |  |
| BRESU_RS00470 | Bresu_0092 | hypothetical protein | 1 | 17 | yes | yes | yes |  |
| BRESU_RS02585 | Bresu_0517 | two-component sensor DivL | 2 | -13 | yes | no |  |  |
| BRESU_RS03120 | Bresu_0624 | GcrA cell cycle regulator | 3 | 19 | yes | no |  |  |
| BRESU_RS03340 | Bresu_0668 | hypothetical protein | 1 | 16 | yes | no |  |  |
| BRESU_RS04425 | Bresu_0887 | arsenate reductase (glutaredoxin) | 2 | -10 | yes | yes | BRESU_RS04410 |  |
| BRESU_RS04745 | Bresu_0953 | extensin | 1 | 15 | yes | no |  |  |
| BRESU_RS05470 | Bresu_1100 | HNH endonuclease | 1 | 90 | yes | no |  |  |
| BRESU_RS06580 | Bresu_1330 | GGDEF protein DgcA | 1 | 32 | yes | no |  |  |
| BRESU_RS06755 | Bresu_1366 | hypothetical protein | 1 | 39 | yes | no |  |  |
| BRESU_RS07120 | Bresu_1445 | SciP | 1 | -71 | yes | no |  |  |
| BRESU_RS07780 | Bresu_1578 | MipZ | 2 | 29 | yes | yes | yes |  |
| BRESU_RS07785 | Bresu_1579 | molecular chaperone DnaJ | 2 | 29 | yes | yes | BRESU_RS07780 |  |
| BRESU_RS08130 | Bresu_1647 | limonene-1,2-epoxide hydrolase | 2 | 39 | yes | no |  |  |
| BRESU_RS08815 | Bresu_1779 | spermidine synthase | 1 | 55 | yes | no |  |  |
| BRESU_RS09235 | Bresu_1865 | DNA topoisomerase IV subunit B | 2 | -15 | yes | yes | BRESU_RS09240 |  |
| BRESU_RS09250 | Bresu_1868 | 2,5-didehydrogluconate reductase | 0 |  | yes | no |  | One GANTC site located -191 bp down from start codon |
| BRESU_RS09825 | Bresu_1979 | DNA topoisomerase IV subunit A | 1 | 13 | yes | no |  |  |
| BRESU_RS09865 | Bresu_1987 | hypothetical protein | 1 | 14 | yes | no |  |  |
| BRESU_RS09945 | Bresu_2002 | hypothetical protein | 4 | 25 | yes | yes | BRESU_RS09950 |  |
| BRESU_RS09950 | Bresu_2003 | hypothetical protein | 4 | 25 | yes | yes | yes |  |
| BRESU_RS10115 | Bresu_2039 | hypothetical protein | 2 | -54 | yes | no |  |  |
| BRESU_RS11060 | Bresu_2229 | diguanylate phosphodiesterase | 1 | 29 | yes | no |  |  |
| BRESU_RS11765 | Bresu_2369 | MFS transporter | 1 | 24 | yes | yes | yes |  |
| BRESU_RS11770 | Bresu_2370 | nucleoside triphosphate pyrophosphohydrolase | 1 | 24 | yes | yes | BRESU_RS11765 |  |
| BRESU_RS12235 | Bresu_2461 | Hpt domain-containing protein | 1 | 33 | yes | yes | BRESU_RS12240 |  |
| BRESU_RS12240 | Bresu_2462 | amino acid permease | 1 | 33 | yes | yes | yes |  |
| BRESU_RS12335 | Bresu_2477 | phosphoribosyl-ATP pyrophosphohydrolase | 1 | -21 | yes | yes | BRESU_RS12330 |  |
| BRESU_RS13085 | Bresu_2628 | DNA gyrase subunit A | 2 | 89 | yes | yes | yes |  |
| BRESU_RS13720 | Bresu_2751 | gamma carbonic anhydrase family protein | 2 | 80 | yes | no |  |  |
| BRESU_RS13725 | Bresu_2752 | phosphate starvation protein PhoH | 0 |  | yes | no |  | One GANTC site located 223 bp up from start codon |
| BRESU_RS14105 | Bresu_2827 | pyrroline-5-carboxylate reductase | 2 | 38 | yes | yes | BRESU_RS14110 |  |
| BRESU_RS14110 | Bresu_2828 | hypothetical protein | 2 | 38 | yes | yes | yes |  |
| BRESU_RS14540 | Bresu_2914 | hypothetical protein | 1 | 42 | yes | no |  |  |
| BRESU_RS14550 | Bresu_2918 | hypothetical protein | 0 |  | yes | no |  | One GANTC site located 145bp up from start codon |
| BRESU_RS14665 | Bresu_2934 | MFS transporter | 1 | 13 | yes | no |  |  |
| BRESU_RS15075 | Bresu_3016 | CckA | 1 | 19 | yes | no |  |  |
| BRESU_RS15155 | Bresu_3032 | DNA-3-methyladenine glycosylase | 1 | 11 | yes | no |  |  |

**Table G: List of upregulated genes under the direct regulation of GcrA in *B. subvibrioides* (P<0.01, >2-fold and presence of GcrA peak in the promoter region (-100 bp to +100 bp of the translational start site))**

| **Locus Tag** | **Old Locus Tag** | **Description** | **GANTC sites** | **Distance** | **GcrA peak** | **Part of operon** | **First gene of operon** | **Notes** |
| --- | --- | --- | --- | --- | --- | --- | --- | --- |
| BRESU_RS02670 | Bresu_0534 | signal transduction histidine kinase | 1 | 49 | yes | yes | BRESU_RS02675 |  |
| BRESU_RS06905 | Bresu_1398 | hypothetical protein | 1 | -19 | yes | yes | BRESU_RS06900 |  |
| BRESU_RS06615 | Bresu_1337 | DNA-binding protein | 1 | -62 | yes | no |  |  |
| BRESU_RS06820 | Bresu_1380 | peptidase S49 | 0 |  | yes | yes | BRESU_RS06800 | One GANTC site located 292 bp up from start codon of first gene of this operon |
| BRESU_RS02355 | Bresu_0471 | hypothetical protein | 0 |  | yes | no |  | One GANTC site located 218bp up from start codon |
| BRESU_RS03780 | Bresu_0759 | glutathione S-transferase | 0 |  | yes | no |  | One GANTC site located 185bp up from start codon |

**Table H: List of genes common in CcrM and GcrA regulon in *B. subvibrioides* (>2-fold, P<0.01 and GcrA peak with GANTC site in the promoter region).**

| **Locus tag** | **Old locus tag** | **Description** |
| --- | --- | --- |
| BRESU_RS12240 | Bresu_2462 | amino acid permease |
| BRESU_RS15075 | Bresu_3016 | CckA |
| BRESU_RS04825 | Bresu_0969 | cell wall hydrolase |
| BRESU_RS13085 | Bresu_2628 | DNA gyrase subunit A GyrA |
| BRESU_RS09235 | Bresu_1865 | DNA topoisomerase IV subunit B ParE |
| BRESU_RS15155 | Bresu_3032 | DNA-3-methyladenine glycosylase |
| BRESU_RS04745 | Bresu_0953 | extensin |
| BRESU_RS05470 | Bresu_1100 | HNH endonuclease |
| BRESU_RS09865 | Bresu_1987 | hypothetical protein |
| BRESU_RS14540 | Bresu_2914 | hypothetical protein |
| BRESU_RS10115 | Bresu_2039 | hypothetical protein |
| BRESU_RS03340 | Bresu_0668 | hypothetical protein |
| BRESU_RS09950 | Bresu_2003 | hypothetical protein |
| BRESU_RS06755 | Bresu_1366 | hypothetical protein |
| BRESU_RS09945 | Bresu_2002 | hypothetical protein |
| BRESU_RS08130 | Bresu_1647 | limonene-1,2-epoxide hydrolase |
| BRESU_RS11765 | Bresu_2369 | MFS transporter |
| BRESU_RS14665 | Bresu_2934 | MFS transporter |
| BRESU_RS08815 | Bresu_1779 | spermidine synthase |

**Table I: List of genes common in CcrM and GcrA regulon in C*. crescentus*. 204 genes belonging to GcrA regulon (Haakonsen et al (2015)) were compared with 78 genes belonging to CcrM regulon (>2 fold, GANTC site in promoter) (Gonzalez et al (2014)).**

| CCNA_00157 | glyoxalase family protein |
| --- | --- |
| CCNA_00861 | hypothetical protein |
| CCNA_00869 | DNA modification methyltransferase-like protein |
| CCNA_01204 | dTDP-glucose 4,6-dehydratase |
| CCNA_01258 | permease |
| CCNA_01268 | cytochrome c |
| CCNA_01450 | single-stranded-DNA-specific exonuclease RecJ |
| CCNA_01542 | ice nucleation protein |
| CCNA_01590 | NAD-dependent DNA ligase |
| CCNA_01624 | orotate phosphoribosyltransferase |
| CCNA_01647 | 8-amino-7-oxononanoate synthase |
| CCNA_01651 | DNA gyrase subunit A |
| CCNA_01778 | penicillin-binding protein |
| CCNA_01914 | 16S rRNA m(2)G 1207 methyltransferase |
| CCNA_01978 | methyltransferase |
| CCNA_02005 | inosine-uridine preferring nucleoside hydrolase |
| CCNA_02052 | DNA topoisomerase IV subunit B |
| CCNA_02086 | sporulation domain-containing protein |
| CCNA_02091 | hypothetical protein |
| CCNA_02127 | oxacillin resistance-associated protein fmtC |
| CCNA_02208 | thymidylate synthase |
| CCNA_02260 | hypothetical protein |
| CCNA_02281 | membrane alanine aminopeptidase |
| CCNA_02363 | hypothetical protein |
| CCNA_02395 | hypothetical protein |
| CCNA_02401 | transcriptional regulator |
| CCNA_02416 | DNA-binding protein HU |
| CCNA_02622 | M61 glycyl aminopeptidase |
| CCNA_02623 | cell division protein FtsZ |
| CCNA_02679 | hypothetical protein |
| CCNA_02710 | membrane protein |
| CCNA_03127 | aminoacyl-histidine dipeptidase |
| CCNA_03408 | ABC transporter ATP-binding protein |

**Table J: List of common genes under direct regulation of GcrA in *C. crescentus* (Haakonsen et al (2015)) and *B. subvibrioides* (P<0.01, and presence of GcrA peak in the promoter region (-100 bp to +100 bp of the translational start site).**

| **Gene name** | **Description** | ***C. crescentus* gene** | **B*. subvibrioides* gene** |
| --- | --- | --- | --- |
| *cckA* | sensory transduction histidine kinase/receiver protein | CCNA_01132 | Bresu_3016 |
| *mipZ* | division plane positioning ATPase | CCNA_02246 | Bresu_1578 |
| *parE* | DNA topoisomerase IV subunit B | CCNA_02052 | Bresu_1865 |
| *tipF* | cyclic-di-GMP phosphodiesterase | CCNA_00747 | Bresu_2229 |
| *parC* | DNA topoisomerase IV subunit A | CCNA_01637 | Bresu_1979 |
| *gyrA* | DNA gyrase subunit A | CCNA_01651 | Bresu_2628 |
| *divL* | two-component sensor histidine kinase | CCNA_03598 | Bresu_0517 |
| *ftsZ* | cell division protein | CCNA_02623 | Bresu_2686 |
|  | hypothetical protein | CCNA_00527 | Bresu_2828 |
| *pleC* | sensory transduction histidine kinase | CCNA_02567 | Bresu_0892 |
| *dgcA* | Diguanylate cyclase | CCNA_03394 | Bresu_1330 |
| *flbA* | Flagellar protein  TPR repeat-containing  protein | CCNA_01524 | Bresu_2406 |
| *thyA* | thymidylate synthase | CCNA_02208 | Bresu_2476 |
| *xseA* | exodeoxyribonuclease VII large subunit | CCNA_02329 | Bresu_1846 |
|  | M16 family peptidase | CCNA_02721 | Bresu_2748 |
| *gyrB* | DNA gyrase subunit B | CCNA_00159 | Bresu_0006 |
|  | thioredoxin-disulfide reductase | CCNA_02964 | Bresu_0978 |
|  | aminopeptidase | CCNA_02588 | Bresu_1628 |
| *ftsN* | Cell division protein | CCNA_02086 | Bresu_2988 |
|  | anhydromuramoyl-peptide exo-beta-N-acetylglucosaminidase | CCNA_02085 | Bresu_2987 |
| *ligA* | NAD-dependent DNA ligase | CCNA_01590 | Bresu_1627 |
| *pmbA* | microcin-processing peptidase | CCNA_02915 | Bresu_2430 |
| *cpaC* | outer membrane pilus secretion channel cpaC | CCNA_03040 | Bresu_0641 |
| *phoR* | phosphate regulon sensor protein | CCNA_00291 | Bresu_1698 |

**Table K: List of common genes misregulated in *gcrA* mutant in *C. crescentus* and *B. subvibrioides* (misregulated genes from Holtzenhoff *et al*. (2004) was compared to the list obtained from this study).**

| **Gene name** | **Description** | ***C. crescentus* gene** | ***B. subvibrioides* gene** |
| --- | --- | --- | --- |
|  | Hypothetical protein | CCNA_01386 | Bresu_1825 |
| *parE* | DNA topoisomerase IV subunit B | CCNA_02052 | Bresu_1865 |
| *gyrA* | DNA gyrase subunit A | CCNA_01651 | Bresu_2628 |
| *tipF* | diguanylate phosphodiesterase | CCNA_00747 | Bresu_2229 |
|  | Hypothetical protein | CCNA_00527 | Bresu_2828 |

**References:**

Curtis PD, Brun Y V. Identification of essential alphaproteobacterial genes reveals operational variability in conserved developmental and cell cycle systems. Mol Microbiol. 2014;93: 713–735. doi:10.1111/mmi.12686

Gober, JW and Shapiro L. A developmentally regulated *Caulobacter* flagellar promoter is activated by 3’ enhancer and IHF binding elements. Mol. Biol. Cell. 1992;3: 913–926.

Haakonsen DL, Yuan AH, Laub MT. The bacterial cell cycle regulator GcrA is a σ70 cofactor that drives gene expression from a subset of methylated promoters. Genes Dev. 2015;29: 2272–2286.

Gonzalez D, Kozdon JB, McAdams HH, Shapiro L, Collier J. The functions of DNA methylation by CcrM in *Caulobacter crescentus*: A global approach. Nucleic Acids Res. 2014;42: 3720–3735.
